# Targeting SIRT3 dysfunction with a sprayable honokiol hydrogel for diabetic burn wound repair

**DOI:** 10.3389/fphar.2026.1912136

**Published:** 2026-09-08

**Authors:** Min Li, Xuedong Yu, Huantao Hu, Kai Wen, Qiongfang Ruan, Paul Yao, Maolong Dong

**Affiliations:** 1 Department of Burns, Wuhan Third Hospital & Tongren Hospital of Wuhan University, Wuhan, China; 2 Hubei Provincial Clinical Medical Research Center for Burns, Wuhan, China; 3 Equine Science Research and Horse Doping Control Laboratory, School of Physical Education, Wuhan Business University, Wuhan, China

**Keywords:** diabetic burn wound, honokiol, mitochondrial oxidative stress, Nrf2, SIRT3, wound healing

## Abstract

**Background:**

Diabetic burn wound healing is impaired by persistent mitochondrial oxidative stress, chronic inflammation, bacterial infection, and defective tissue regeneration. Although honokiol possesses antioxidant and anti-inflammatory properties, its therapeutic potential is limited by poor local retention. Moreover, the therapeutic significance of restoring mitochondrial sirtuin 3 (SIRT3) signaling in diabetic wound repair remains largely unexplored. This study developed a sprayable honokiol-loaded catechol-chitosan hydrogel as a mechanism-guided therapeutic strategy for diabetic burn wounds.

**Methods:**

Two hydrogel formulations with distinct crosslinking densities were engineered using catechol-modified chitosan and oxidized dextran to achieve tunable adhesion and honokiol release profiles. Physicochemical properties, drug release, antioxidant and antimicrobial activities, and cytocompatibility were evaluated. Mechanistic studies were conducted in hyperglycemia-challenged endothelial cells and macrophages, followed by therapeutic assessment in streptozotocin-induced diabetic burn wound models.

**Results:**

The hydrogels exhibited rapid *in situ* gelation, strong tissue adhesion, sustained honokiol release, favorable biocompatibility, antioxidant activity, and antimicrobial protection. Mechanistically, honokiol hydrogel treatment restored hyperglycemia-induced SIRT3 suppression, improved mitochondrial redox homeostasis, enhanced Nrf2 antioxidant signaling, and attenuated NFκB p65/MMP9-mediated inflammatory and matrix-degrading responses. These effects were associated with reduced oxidative stress and bacterial burden, enhanced reparative macrophage polarization, increased angiogenesis and granulation tissue formation, and accelerated wound closure. The sustained-release formulation demonstrated superior therapeutic efficacy.

**Conclusion:**

This study identifies SIRT3 dysfunction as a therapeutically actionable mechanism in diabetic burn wound healing and demonstrates that sustained local delivery of honokiol effectively restores mitochondrial and inflammatory homeostasis. By simultaneously targeting oxidative stress, inflammation, infection, and impaired regeneration, this sprayable hydrogel platform represents a promising translational pharmacological strategy for diabetic wound management.

## Introduction

Chronic wounds represent a major complication of diabetes and impose significant clinical and socioeconomic burdens worldwide (Armstrong et al., 2017). Persistent hyperglycemia disrupts the normal wound-healing process by inducing oxidative stress, mitochondrial dysfunction, impaired angiogenesis, and prolonged inflammation (Brem and Tomic-Canic, 2007; Falanga, 2005; Xie et al., 2021). These pathological changes compromise critical cellular functions, including keratinocyte migration, fibroblast proliferation, collagen deposition, and extracellular matrix (ECM) remodeling, ultimately delaying tissue regeneration (Gurtner et al., 2008). Furthermore, diabetic wounds are highly susceptible to microbial invasion, and infection exacerbates tissue damage, prolongs the inflammatory phase, and increases the risk of severe complications, such as diabetic foot ulcers and limb amputation (Lipsky et al., 2012). Current therapeutic strategies -including debridement, antibiotics, and advanced wound dressings -provide limited clinical benefit due to the complex wound microenvironment characterized by persistent inflammation, excessive reactive oxygen species (ROS), and poor vascularization (Jeffcoate and Harding, 2003). Accordingly, there is a critical need to develop multifunctional therapeutic strategies capable of simultaneously reducing oxidative stress, controlling inflammation, preventing infection, and promoting tissue regeneration to improve diabetic wound outcomes (Singer and Clark, 1999; Xie et al., 2022).

Hydrogels have gained considerable attention as effective platforms for the treatment of chronic wounds since they maintain a moist environment, provide physical protection, and enable localized, sustained drug delivery (Bowman et al., 2008). However, most clinically available hydrogel systems act primarily as passive dressings and lack the capacity to actively modulate the multifactorial pathological processes underlying diabetic wounds, including oxidative stress, persistent inflammation, and microbial colonization (Xiang et al., 2024; Kolipaka et al., 2024). Incorporation of bioactive compounds into hydrogel matrices offers a promising strategy to enhance therapeutic efficacy (Xiang et al., 2024; Deng et al., 2022). Honokiol, a naturally occurring biphenolic compound derived from Magnolia officinalis, exhibits potent antioxidant, anti-inflammatory, and antimicrobial properties and has demonstrated protective effects in vascular and metabolic disease models (Rauf et al., 2021; Park et al., 2004; Ou et al., 2006; Kim and Jung, 2019; Sun et al., 2026). By integrating honokiol into a hydrogel delivery system, it is possible to achieve sustained local release while simultaneously attenuating oxidative stress, suppressing inflammatory signaling, limiting bacterial growth, and promoting tissue regeneration (Wei et al., 2023). Therefore, honokiol-loaded hydrogels represent a potentially effective multifunctional strategy for improving healing in diabetic wounds.

Mitochondrial dysfunction and redox imbalance are central contributors to impaired wound healing in diabetes, and the mitochondrial deacetylase SIRT3 has emerged as a critical regulator of these processes (Boniakowski et al., 2019). SIRT3 maintains cellular redox homeostasis, supports mitochondrial metabolism, and modulates inflammatory signaling, functions that are frequently disrupted under diabetic conditions (Zheng et al., 2023). Reduced SIRT3 expression in diabetes leads to mitochondrial dysfunction, excessive ROS production, and activation of inflammatory pathways, collectively impairing tissue repair (Yang et al., 2020). Reactivation of SIRT3 has thus been suggested as a potential therapeutic approach to mitigate oxidative stress, enhance mitochondrial resilience, and accelerate wound healing (Cimen et al., 2010). Notably, honokiol has been reported to upregulate SIRT3 expression and improve mitochondrial function, suggesting that honokiol-based therapies may exert protective effects by reactivating SIRT3-dependent pathways (Yu et al., 2025; Shi et al., 2024). Targeting SIRT3 with bioactive molecules such as honokiol may thus provide a promising avenue to restore mitochondrial homeostasis and facilitate tissue repair under diabetic conditions.

To address the multifactorial barriers to diabetic wound repair, we developed a sprayable bioadhesive hydrogel platform based on catechol-modified chitosan and oxidized dextran for localized delivery of honokiol (Liu et al., 2022). Unlike conventional honokiol-loaded dressings that primarily serve as passive drug carriers, this study employed a mechanism-driven design strategy that integrates tunable hydrogel architecture with targeted modulation of key pathogenic pathways involved in diabetic wound healing. Two hydrogel formulations with distinct crosslinking densities were engineered to achieve differential release kinetics, enabling direct evaluation of how sustained local honokiol delivery influences therapeutic outcomes. We hypothesized that controlled activation of the SIRT3-centered redox regulatory network would simultaneously attenuate mitochondrial oxidative stress, chronic inflammation, infection susceptibility, impaired angiogenesis, and abnormal extracellular matrix remodeling. Specifically, we investigated whether honokiol-loaded hydrogels could restore hyperglycemia-induced SIRT3 suppression, activate Nrf2-dependent antioxidant defenses, inhibit NFκB p65 and MMP9 signaling, promote reparative macrophage polarization, and enhance vascular and tissue regeneration. Through comprehensive physicochemical characterization, mechanistic studies in hyperglycemia-challenged endothelial cells and macrophages, and therapeutic evaluation in a diabetic burn wound model, we sought to establish a multifunctional hydrogel system that not only provides sustained drug release and strong tissue adhesion but also targets the previously underexplored SIRT3–Nrf2–p65–MMP9 signaling axis as a coordinated therapeutic strategy for diabetic wound management.

## Materials and methods

Further methodological details are provided in Supplementary Data S1, and the sequences of primers employed in the study are summarized in Supplementary Table S1.

### Reagents and materials

Antibodies for iNOS (ab115819), MMP9 (ab73734), Nrf2 (ab137550) and SIRT3 (ab217319) were purchased from Abcam. Antibodies for NFκB p65 (#8242) and Histone H3 (#9715) were purchased from Cell Signaling Technology. The 8-oxo-dG antibody (4354-MC-050) was obtained from Novus Biologicals. NFκB p65 activity was measured using the TransAM® NFκB p65 Chemi Kit (#40597; Active Motif) according to the manufacturer’s instructions. The chemiluminescence signal was normalized to the control group and presented as relative activity in arbitrary units (AU). Catechol-modified chitosan (2% w/v, 150–200 kDa, >90% deacetylated) was prepared from medical-grade chitosan (Qingdao Yunzhou Biochemistry Co., Ltd, China) by dopamine conjugation, and oxidized dextran (70 kDa) was generated by sodium periodate oxidation of dextran (Sigma, China). Honokiol (≥98% purity, Sigma) was used as the bioactive compound, and formulation solvents including propylene glycol and medium-chain triglycerides (MCT oil), were obtained from Sigma, China.

### Hydrogel preparation

Catechol-modified chitosan (2% w/v; degree of deacetylation >90%; MW 150–200 kDa) was dissolved in 0.1 M acetic acid under magnetic stirring (300 rpm) for 2 h until a clear, homogeneous solution was obtained. The pH was adjusted to 6.5 using 1M NaOH with continuous monitoring to prevent local precipitation.

Oxidized dextran (MW ∼70 kDa; target oxidation ∼30%) was synthesized by dissolving dextran (10.0 g) in 100.0 mL deionized water, followed by oxidation with sodium periodate (1.50 g) at 25 °C for 2 h in the dark. The reaction was quenched with ethylene glycol (2.00 mL), and the product was dialyzed (MWCO 3.5 kDa) against deionized water at 4 °C for 48 h (medium refreshed every 6 h), then lyophilized to obtain oxidized dextran powder.

Honokiol (≥98% purity, Sigma-Aldrich) was dissolved in DMSO to prepare a concentrated stock solution (100 mg/mL). The stock solution was added dropwise to the catechol-chitosan solution under gentle stirring (100 rpm), yielding a final honokiol concentration of 1.0 mg/mL (0.10% w/v). For a 10.0 mL formulation, this corresponds to 10.0 mg honokiol introduced via 0.100 mL stock solution, resulting in ∼1% (v/v) DMSO in the final mixture. No visible precipitation was observed upon dilution.

Hydrogels were formed via dynamic Schiff-base crosslinking between amine groups of chitosan and aldehyde groups of oxidized dextran. Two crosslinking densities were prepared by adding oxidized dextran to 10.0 mL of the catechol-chitosan/honokiol solution: Hydrogel A (moderate crosslinking, 120 mg) and Hydrogel B (dense crosslinking, 180 mg). Control hydrogels were prepared identically without honokiol (see details in Supplementary Figure S1).

Gelation was initiated at room temperature (22 °C–25 °C) under sterile conditions by mixing the components and gently vortexing for 10 s, with gelation occurring as determined by vial inversion. Rheological properties were evaluated using oscillatory rheological measurements at 25 °C. Storage modulus (G′) and loss modulus (G″) were recorded under frequency sweep conditions (0.1–10 Hz, 1% strain). The hydrogel exhibited a dominant elastic response, with G′ consistently exceeding G″ across the tested frequency range, indicating stable network formation.

Hydrogels were homogeneous with no visible phase separation and were stored at 4 °C under aseptic conditions until use. The selected honokiol concentration was based on prior reports of its efficacy in hydrogel systems and established topical tolerability, while maintaining low DMSO content (∼1% v/v) (Wei et al., 2023; Li et al., 2022).

### Sprayability

To evaluate sprayability, 5 mL of hydrogel was loaded into sterile syringes fitted with 25G needles. The hydrogel was sprayed onto sterile Petri dishes placed 10 cm away. Extrusion force was quantified by pressing the syringe plunger against an analytical balance and recording the maximum applied force required to initiate and sustain flow. Consistent spray patterns and manageable extrusion force were used as indicators of suitability for topical delivery.

### Gelation

Gelation time was determined using the vial inversion method. Aliquots (1 mL) of freshly mixed hydrogel were placed into sterile glass vials at room temperature. The vials were inverted at 10 s intervals, and gelation was confirmed when no visible flow was observed within 1 min. The reproducibility of gelation was confirmed in triplicate for each preparation.

### Adhesion

Adhesive strength was quantified using *ex vivo* porcine dermis as a tissue substrate. Strips of dermis (1 × 2 cm) were cleaned and gently blotted dry. A 100 µL aliquot of hydrogel was applied between two dermis strips, which were then pressed together under a constant weight of 200 g for 2 min to allow bonding. The bonded samples were subsequently subjected to incremental weights until detachment occurred, and the maximum weight sustained before separation was recorded as the detachment force.

### Stability

Hydrogel formulations were aliquoted into sterile tubes and stored at either 4 °C or 37 °C for up to 14 days. Samples were visually inspected on days 0, 7, and 14 for phase separation, turbidity, or color change. Stability was further assessed by measuring OD600 of hydrogel extracts in PBS to monitor aggregation or degradation over time. A formulation was considered stable if no phase separation and less than 10% change in OD600 were observed over the storage period.

### Drug release

Honokiol release was evaluated using a dialysis method. Samples (1 mg honokiol) were placed in dialysis tubing (MWCO 3.5 kDa) and incubated in PBS (pH 7.4) at 37 °C with shaking. At defined time points, aliquots were collected and replaced with fresh PBS. Honokiol concentration was measured by UV–Vis at 290 nm.

### Antioxidant activity

Radical scavenging activity was assessed by DPPH assay. Samples were mixed with 0.1 mM DPPH, incubated in the dark for 30 min, and absorbance was read at 517 nm. $\text{Scavenging }\left(\%\right)=\left[\left(A\_\text{control}-A\_\text{sample}\right)/A\_\text{control}\right]\times 100$ .

### Antibacterial activity (agar diffusion)

Methicillin-Resistant *Staphylococcus aureus* (MRSA), *Pseudomonas aeruginosa* (PA), and *Escherichia coli* (Ecoli) (∼10^6^ CFU/mL) were spread on agar plates. Discs loaded with hydrogel samples were applied and incubated at 37 °C for 24 h. Inhibition zones were measured.

### Minimum inhibitory concentration (MIC)

MIC was determined by broth microdilution. Serially diluted samples were incubated with bacteria (10^5^ CFU/mL) for 24 h. OD_600_ was measured, and MIC was defined as the lowest concentration with no significant growth (≤0.05 above control).

### Cytocompatibility

HaCaT, NIH 3T3, and HUVECs were treated with extracts for 24 h. Cell viability was assessed by MTT assay (570 nm), with ≥80% viability considered acceptable.

### Oxidative stress measurements

ROS production was assessed using CM-H_2_DCFDA (10 μM; Invitrogen) with fluorescence recorded at 485/530 nm after 45 min incubation at 37 °C. Oxidative DNA damage (8-oxo-dG) was quantified by immunofluorescence and ImageJ analysis. Cellular redox status (GSH/GSSG) was measured using the GSH/GSSG-Glo™ kit (Promega). Nrf2 transcriptional activity was measured using the Nrf2 Transcription Factor Assay Kit (Colorimetric; Abcam, #ab207223). Absorbance values were normalized to the control group (set as 100%) and presented as relative Nrf2 DNA-binding activity. Methylglyoxal (MG) levels were quantified using the Methylglyoxal Assay Kit (Abcam, #ab241006), calculated from the manufacturer’s standard curve, and presented as concentrations (Wang et al., 2019).

#### *In vivo* mouse studies

All procedures involving injury or surgery were performed under inhalational anesthesia, and appropriate perioperative and postoperative analgesia was administered in accordance with institutional veterinary guidelines. Euthanasia and tissue collection were carried out following AVMA-approved protocols.

### Streptozotocin-induced diabetic model

Male C57BL/6 mice (8–10 weeks old) were allowed to acclimate for a minimum of 1 week before use and were housed at 3–5 animals per cage. Diabetes was established via intraperitoneal administration of streptozotocin (STZ, 50 mg/kg) for five consecutive days after a 6–8 h fasting period. Control animals received equivalent volumes of citrate buffer alone. Blood glucose levels were measured under non-fasting conditions at 7 and 14 days after the final injection. Animals with blood glucose levels exceeding 250 mg/dL on two consecutive readings were considered diabetic and enrolled in subsequent experiments.

### Burn wound and infection model

Mice were anesthetized using isoflurane, delivered at 3%–4% for induction and maintained at 1%–2% throughout the procedure and treated with analgesics as required. The dorsal area was shaved and sterilized, and a full-thickness burn injury (2 cm in diameter) was generated through a pre-heated copper device (75 °C applied for 15 s). To establish an infected wound model, a subset of animals received topical inoculation of methicillin-resistant *S. aureus* (MRSA; 10^6^CFU in 10 µL PBS) immediately after injury. Animals were randomly allocated into four experimental groups (n = 8): non-diabetic control (CTL/VEH), diabetic untreated (STZ/VEH), diabetic treated with Hydrogel A (STZ/Hydrogel A), and diabetic treated with Hydrogel B (STZ/Hydrogel B).

### Treatment protocol

Hydrogel formulations were applied topically to wounds at a volume of 50 µL per site under aseptic conditions and covered with a semi-occlusive Tegaderm dressing. Treatments were administered once daily for a total of 21 days. Control animals were treated with an equal volume of the vehicle hydrogel. Wounds were inspected daily for signs of irritation, infection, or adverse responses, and dressings were replaced concurrently with each application.

### Evaluation of wound healing and tissue analysis

Wound progression was documented by digital imaging every 2 days over a 21-day period. Wound size was quantified relative to the initial area using ImageJ. At designated time points, wound tissues were collected and either rapidly frozen or fixed for subsequent histological evaluation, including hematoxylin and eosin (H&E) staining and immunohistochemical analysis. H&E-stained sections were scanned using a Carl Zeiss MIRAX MIDI whole-slide imaging system, and tissue morphology and granulation tissue formation were evaluated using 3DHISTECH Pannoramic Viewer software. Granulation tissue area and formation were quantitatively analyzed based on whole-slide images to assess the extent of tissue regeneration among different treatment groups. Neovascularization was evaluated by immunohistochemical staining for CD31 on frozen tissue sections. Representative images were acquired at 200× magnification, and CD31-positive cells were quantified in five randomly selected high-power fields (HPFs) per tissue section by a blinded investigator. The average number of CD31-positive cells per HPF was used for statistical analysis. Cytokine concentrations (TNFα, IL6, IL10, and TGFβ) were measured in wound tissue homogenates and plasma samples using ELISA kits (R&D Systems). Gene expression levels of MMP9, NFκB p65, Nrf2, and SIRT3 were analyzed in wound tissues. Oxidative status was evaluated by analyzing Nrf2 activity, the ratio of reduced to oxidized glutathione (GSH/GSSG), and levels of 8-oxo-dG. Antibacterial efficacy was assessed by homogenizing wound tissues, preparing serial dilutions, and plating on mannitol salt agar. Colonies of Methicillin-resistant *S. aureus* were counted as colony-forming units (CFUs) following incubation at 37 °C for 24 h. Systemic exposure to honokiol following topical application was determined using LC–MS/MS, and pharmacokinetic profiles were analyzed in a separate group of animals (Zhang et al., 2018; Thangarajah et al., 2009).

### Bone marrow–derived macrophage (BMM) culture and polarization

Bone marrow cells from C57BL/6 mice were isolated, filtered, and subjected to red blood cell lysis, then cultured in RPMI-1640 with 10% FBS, antibiotics, and M-CSF (20–30 ng/mL). Medium was partially refreshed on day 3, and adherent macrophages were collected on day 7. Cells were replated and polarized to M0, M1 (LPS + IFNγ), or M2 (IL-4) for 18–24 h. Supernatants were analyzed for cytokines (TNFα, IL6, IL10, TGFβ). NFκB activation was assessed by immunoblotting of phospho-p65 and total p65 after short-term stimulation.

### Plasma honokiol quantification

Pharmacokinetics were evaluated in mice (n = 5/group). Plasma collected 0.5–24 h after treatment was extracted with acetonitrile containing internal standard, dried, and reconstituted. Honokiol levels were measured by LC–MS/MS (C18 column, negative ESI, MRM) using calibration standards (10–5,000 ng/mL; LLOQ 10 ng/mL) to generate concentration–time profiles.

### Cytokine measurement by ELISA

Cytokines (TNFα, IL6, IL10, TGFβ) were determined by commercial ELISA kits (R&D Systems). Supernatants collected at 18–24 h were clarified (300 × g, 5 min) and analyzed or stored at −80 °C. Standards were serially diluted to generate calibration curves. Samples (100 μL, duplicates) were incubated in pre-coated plates, followed by detection antibody and HRP–streptavidin incubation. TMB substrate was applied for color development, the reaction was terminated, and absorbance was measured at 450 nm.

## Results

### Honokiol ameliorates hyperglycemia-induced SIRT3 suppression and the associated oxidative stress and inflammatory responses

**FIGURE 1:**
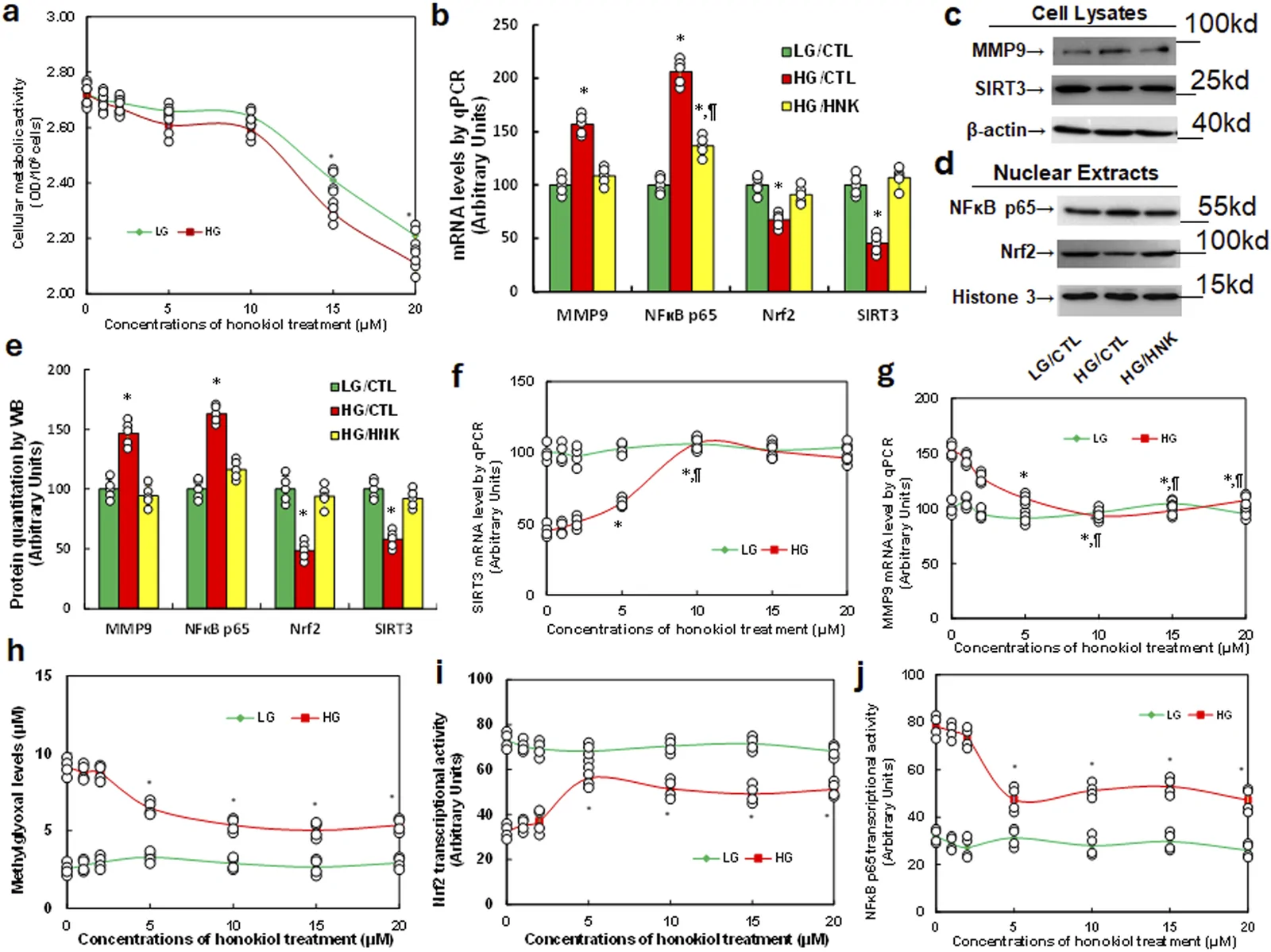
Honokiol ameliorates hyperglycemia-mediated SIRT3 suppression and subsequent oxidative stress and inflammation. **(a)** HUVEC cells were incubated with either low glucose (LG, 5 mM), or high glucose (HG, 25 mM) in the presence of 0–20 µM honokiol for 3 days for cell viability assay, n = 4. *, *P* < 0.05, vs. LG group. **(b–e)** HUVEC cells were incubated with either LG or HG in the presence of 10 µM honokiol (HNK) or empty control (CTL) for 3 days, cells were isolated for gene expression analysis. **(b)** mRNA levels, n = 4. **(c,d)** Representative western blots for cell lysates **(c)** and nuclear extracts **(d)**. **(e)** Protein quantitation for **(c,d)**, n = 5. *, *P* < 0.05, vs. LG/CTL group, *P* < 0.05, vs. HG/CTL group. **(f–j)** Treated HUVEC as shown in **(a)** were used for biological assays. **(f)** SIRT3 mRNA. **(g)** MMP9 mRNA. **(h)** Methylglyoxal formation. **(i)** Nrf2 activity. **(j)** NFκB p65 activity. n = 4. *, *P* < 0.05, vs. 0 µM group, *P* < 0.05, vs. 5 µM group. Data were expressed as mean ± SEM.

To evaluate its effects, HUVECs were maintained under both low-glucose (LG) and high-glucose (HG) conditions. Honokiol showed negligible cytotoxicity at concentrations up to 10 μM, whereas a marked decrease in cellular metabolic activity was observed at 15 µM (Figure 1a). HG exposure markedly increased the mRNA expression of MMP9 and NFκB p65, while significantly decreasing Nrf2 and SIRT3 expression; these alterations were effectively reversed by honokiol treatment (Figure 1b). Consistent trends were observed at the protein level (Figures 1c–e, Supplementary Figure S2). Dose–response analysis further demonstrated that 5 μM honokiol fully restored SIRT3 expression (Figure 1f) and suppressed MMP9 activation (Figure 1g) under HG conditions. Moreover, treatment with 5 μM honokiol partially attenuated HG-induced methylglyoxal accumulation (Figure 1h), restored Nrf2 activity (Figure 1i), and reduced NFκB p65 activity (Figure 1j).

### Honokiol hydrogel formulation and characterization

**FIGURE 2:**
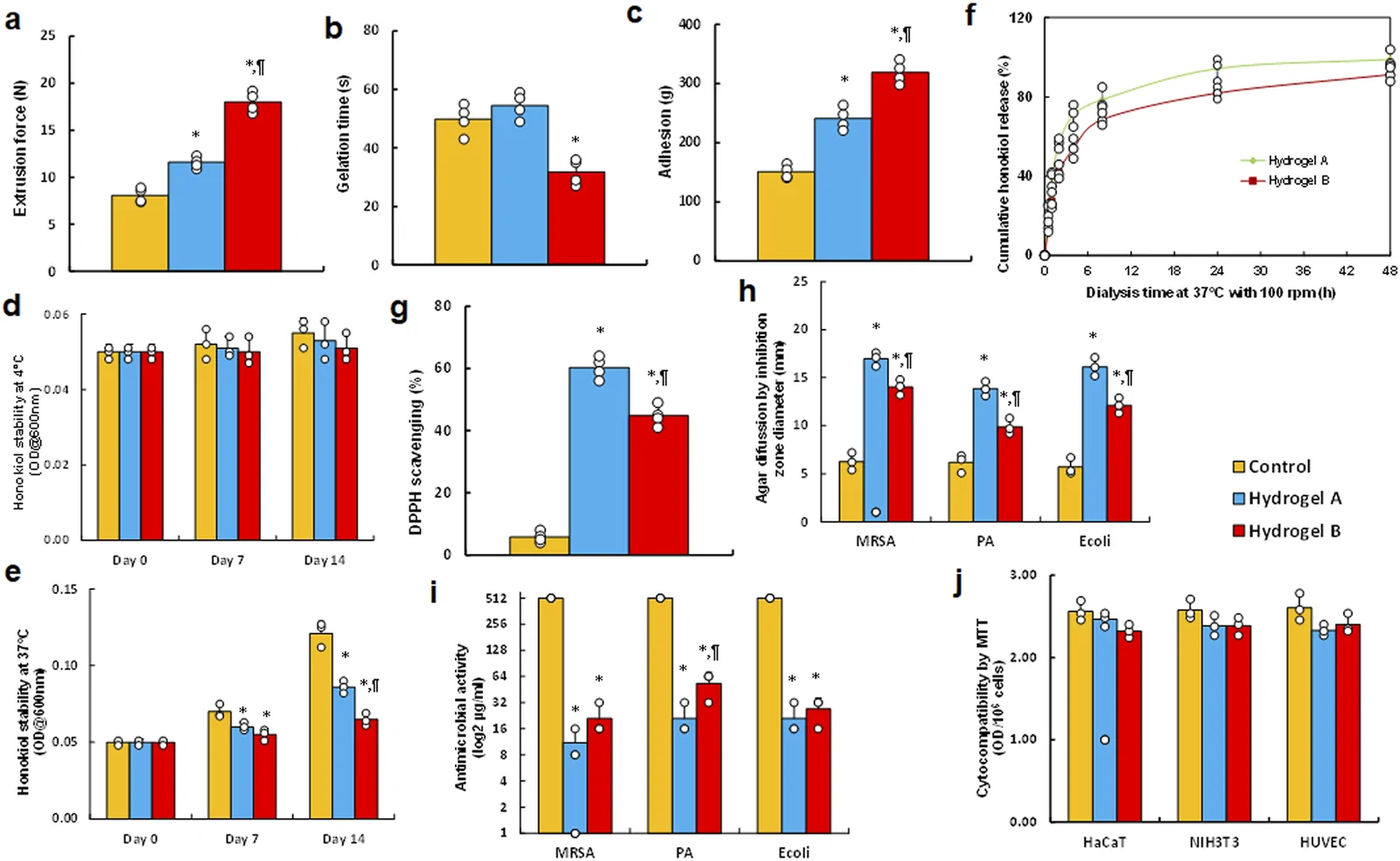
Characterization of honokiol hydrogel. Hydrogel A, Hydrogel B or related Control without honokiol were prepared for characterization of honokiol hydrogel. **(a)** Sprayability. **(b)** Gelation time. **(c)** Adhesion. **(d,e)** Stability at 4 °C **(d)** and 37 °C **(e)** on day 0, 7 and 14. **(f)** Drug release kinetics. **(g)** Antioxidant activity by DPPH scavenging. **(h)** Antimicrobial activity by agar diffusion. **(i)** Antimicrobial activity by broth microdilution. **(j)** Cytocompatibility by MTT. n = 4. *, *P* < 0.05, vs. Control group, *P* < 0.05, vs. Hydrogel A group. Data were expressed as mean ± SEM.

Two honokiol-loaded hydrogels, Hydrogel A (moderate crosslinking) and Hydrogel B (dense crosslinking), and a control hydrogel, were successfully prepared using catechol-modified chitosan and oxidized dextran. All formulations were homogeneous. Increased crosslinking elevated extrusion force and produced thicker filaments (Figure 2a). Gelation was rapid for all groups; Hydrogel A gelled slightly slower, whereas Hydrogel B gelled faster, indicating enhanced network formation at higher crosslinking density (Figure 2b). Both honokiol-loaded hydrogels showed stronger adhesion than the control, with Hydrogel B exhibiting the highest strength, and honokiol incorporation did not impair adhesion (Figure 2c). All formulations were stable at 4 °C but showed reduced stability at 37 °C, where Hydrogel B remained the most stable and the control degraded most (Figures 2d,e). Both hydrogels exhibited sustained drug release, with Hydrogel A showing faster initial release and Hydrogel B demonstrating slower, prolonged release (Figure 2f). Consistently, both hydrogels displayed strong antioxidant activity, with Hydrogel A showing greater DPPH scavenging capacity (Figure 2g). Antimicrobial assays confirmed that both honokiol-loaded hydrogels inhibited MRSA, *P. aeruginosa*, and *E. coli*, with stronger effects observed for Hydrogel A, while the control was inactive (Figures 2h,i). All hydrogel extracts were cytocompatible in HaCaT, NIH 3T3, and HUVEC cells, with only slight reductions in viability in honokiol-loaded groups, particularly Hydrogel B (Figure 2j).

### Honokiol hydrogel attenuates hyperglycemia-induced oxidative stress in HUVECs

**FIGURE 3:**
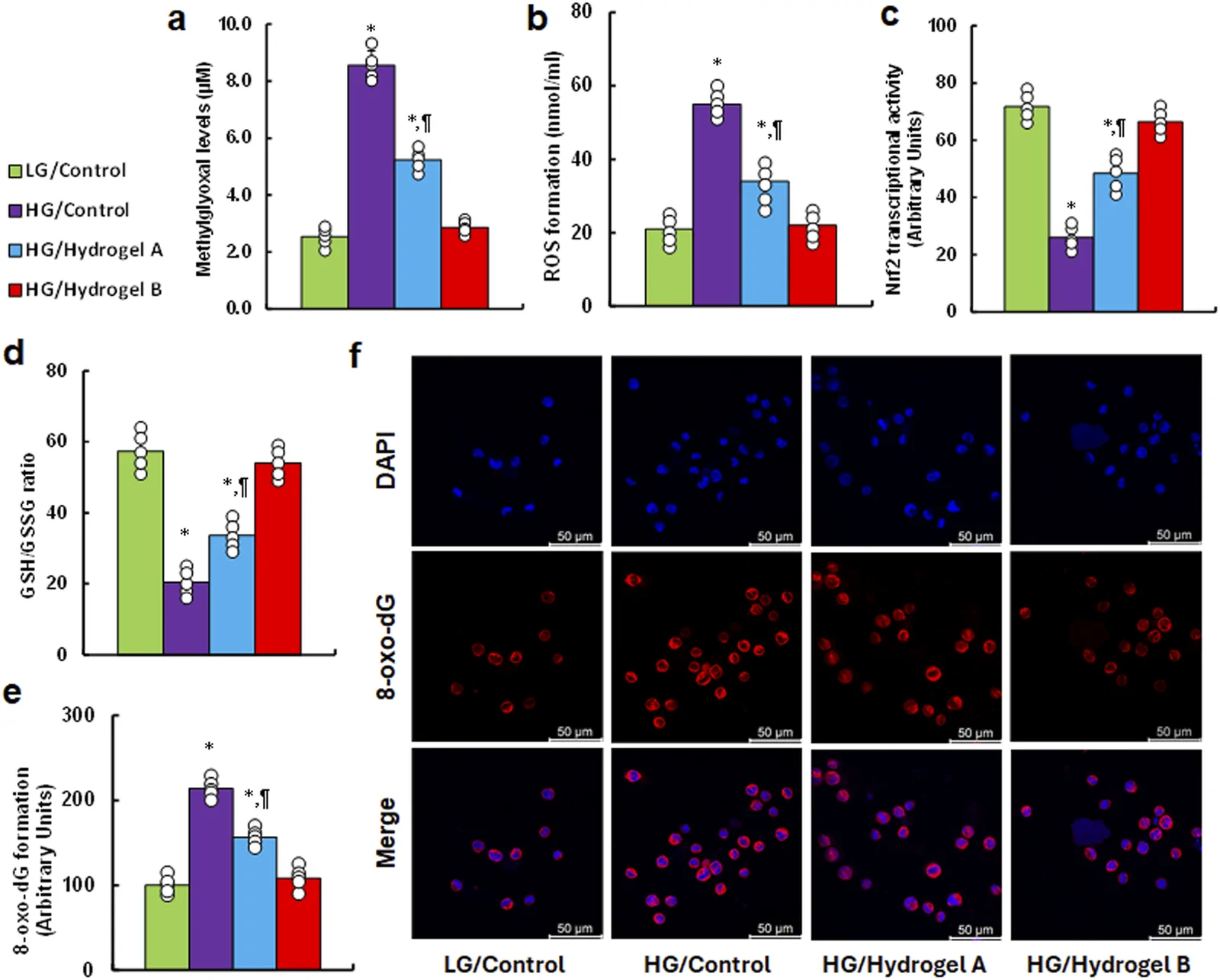
Honokiol Hydrogel ameliorates hyperglycemia-mediated oxidative stress in HUVEC cells. HUVEC cells were incubated with LG, HG, or HG in the presence of control, hydrogel A or hydrogel B for 3 days for analysis of oxidative stress. **(a)** Methylglyoxal formation. **(b)** ROS formation. **(c)** Nrf2 activity. **(d)** GSH/GSSG ratio. **(e)** 8-oxo-dG formation. **(f)** Representative images for **(e)**. n = 5. *, *P* < 0.05, vs. LG/Control group, *P* < 0.05, vs. HG/Control group. Data were expressed as mean ± SEM.

The effects of honokiol hydrogels on oxidative stress under hyperglycemic conditions were evaluated in HUVECs. Compared with normoglycemic controls (LG), hyperglycemia (HG) significantly increased methylglyoxal and ROS production (Figures 3a,b), reduced Nrf2 activity and the GSH/GSSG ratio (Figures 3c,d), and elevated 8-oxo-dG levels (Figures 3e,f). Treatment with Hydrogel A partially, whereas Hydrogel B fully, reversed these HG-induced alterations, indicating a more potent antioxidative effect of the densely crosslinked formulation.

### Honokiol hydrogel restores hyperglycemia-induced mitochondrial dysfunction in HUVECs

**FIGURE 4:**
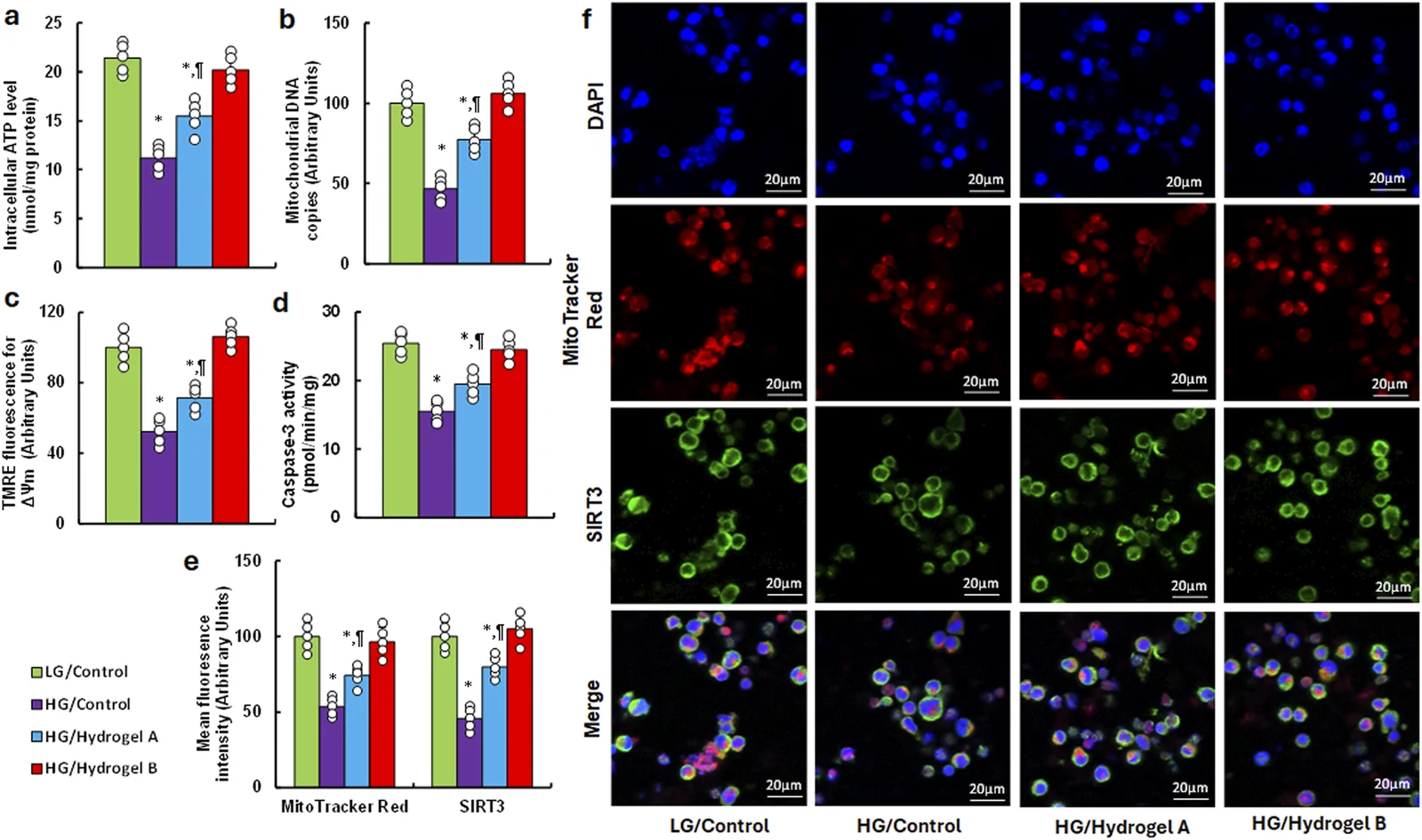
Honokiol Hydrogel ameliorates hyperglycemia-mediated mitochondrial dysfunction in HUVEC cells. HUVEC cells were incubated with LG, HG, or HG in the presence of control, hydrogel A or hydrogel B for 3 days for evaluation of mitochondrial function. **(a)** Intracellular ATP levels. **(b)** Mitochondrial DNA copies. **(c)** Mitochondrial membrane potential (ΔΨm). **(d)** Caspase-3 activity. **(e)** Quantitation of MitoTracker Red and SIRT3. **(f)** Representative images for **(e)**. n = 5. *, *P* < 0.05, vs. LG/Control group, *P* < 0.05, vs. HG/Control group. Data were expressed as mean ± SEM.

The effects of honokiol hydrogels on mitochondrial function under hyperglycemic conditions were assessed in HUVECs. Compared with normoglycemic controls, hyperglycemia significantly reduced ATP production (Figure 4a), mitochondrial DNA copy number (Figure 4b), and mitochondrial membrane potential (ΔΨm) (Figure 4c), while increasing caspase-3 activity (Figure 4d) and decreasing MitoTracker Red and SIRT3 staining intensity (Figures 4e,f). Treatment with Hydrogel A partially, whereas Hydrogel B more effectively, restored these mitochondrial parameters, indicating improved mitochondrial function under hyperglycemic stress.

### Honokiol hydrogel modulates hyperglycemia-induced macrophage polarization and inflammation in BMMs

**FIGURE 5:**
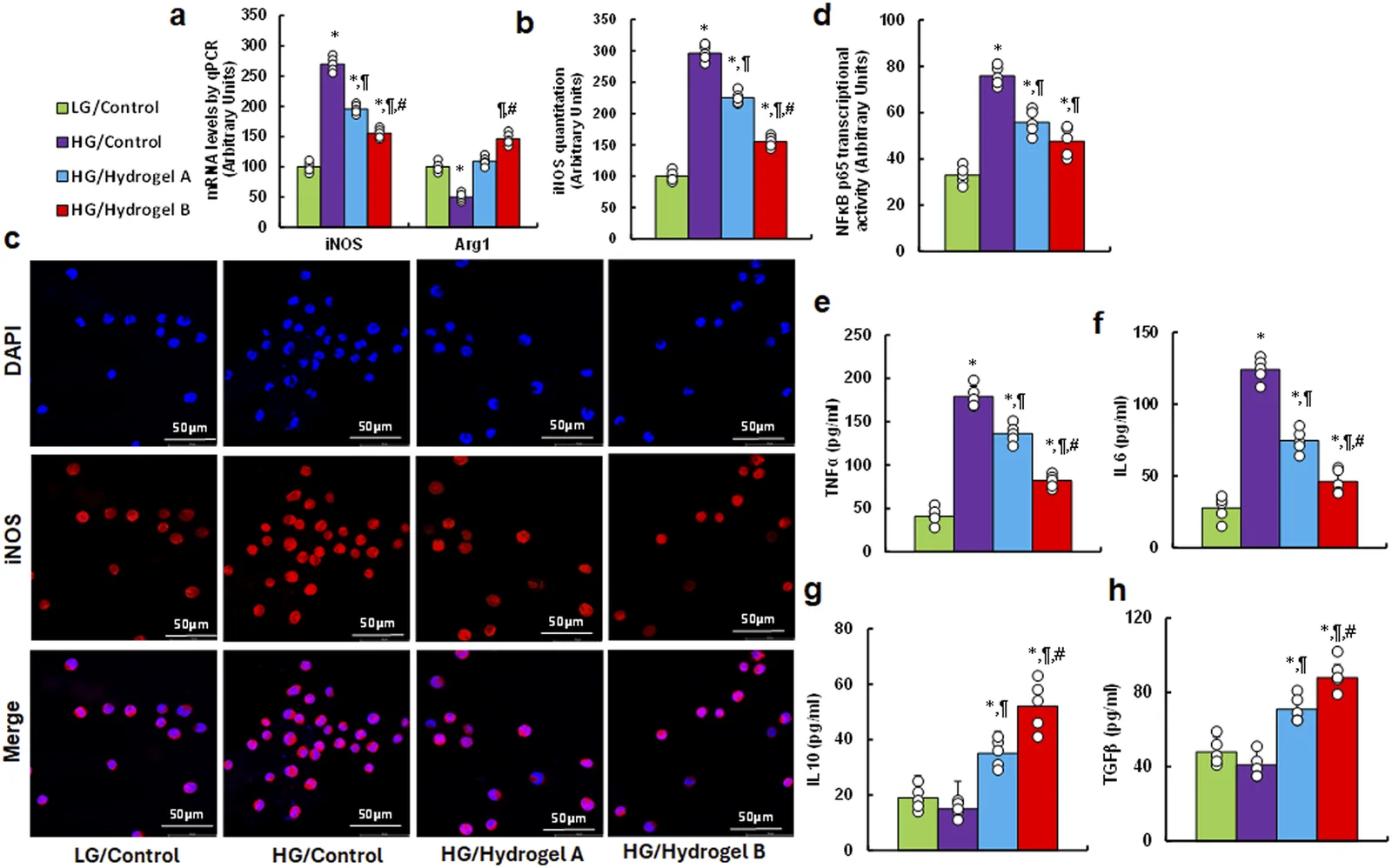
Honokiol Hydrogel ameliorates hyperglycemia-mediated macrophage polarization and inflammation in BMM cells. BMM cells were incubated with LG, HG, or HG in the presence of control, hydrogel A or hydrogel B for 3 days for evaluation of macrophage polarization and inflammation. **(a)** mRNA levels. **(b)** iNOS quantitation by immunostaining. **(c)** Representative images for **(b)**. **(d)** NFκB p65 activity. **(e–h)** Cytokine secretion for TNFα **(e)**, IL6 **(f)**, IL10 **(g)** and TGFβ **(h)**. n = 5. *, *P* < 0.05, vs. LG/Control group, *P* < 0.05, vs. HG/Control group; #, *P* < 0.05, vs. HG/Hydrogel A group. Data were expressed as mean ± SEM.

The effects of honokiol hydrogels on macrophage polarization and inflammatory responses under hyperglycemic conditions were evaluated in BMMs. Compared with normoglycemic controls, hyperglycemia significantly increased iNOS mRNA expression and decreased Arg1 mRNA expression (Figure 5a). Consistent with the RT-qPCR results, immunofluorescence staining demonstrated a marked increase in iNOS expression under hyperglycemic conditions (Figures 5b,c), indicating a polarization toward a pro-inflammatory, M1-like macrophage phenotype. Both Hydrogel A and Hydrogel B partially reversed these changes, with Hydrogel B exhibiting a stronger effect. Hyperglycemia also markedly increased NFκB p65 activity (Figure 5d) and the secretion of TNF-α and IL-6 (Figures 5e,f), which were attenuated by both hydrogels, again with greater efficacy observed for Hydrogel B. In contrast, hyperglycemia had minimal effects on IL10 and TGFβ secretion (Figures 5g,h); however, both hydrogels modestly enhanced their production, with Hydrogel B showing a more pronounced effect.

### Honokiol hydrogel attenuates diabetes- mediated oxidative stress, inflammation, and infection in wound tissues

**FIGURE 6:**
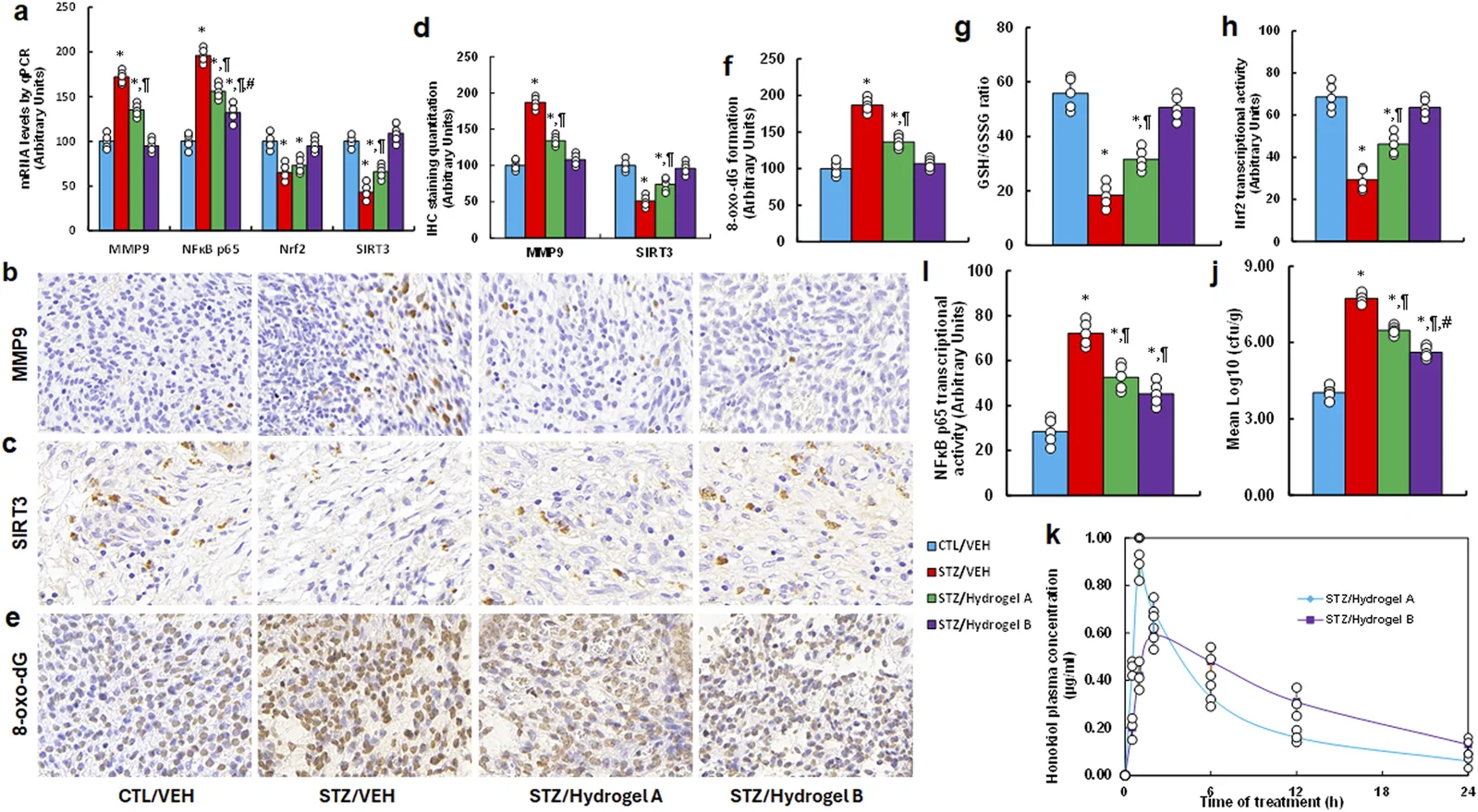
Honokiol hydrogel administration ameliorates diabetes-mediated oxidative stress, inflammation and infection in wound tissues. Mouse models with cutaneous burn injury received CTL or STZ treatment and then received topical administration of vehicle (VEH), Hydrogel A or Hydrogel B, and the wound tissues were then isolated on day 21 after burns for biological assays. **(a)** mRNA levels by qPCR. **(b,c)** Representative images of IHC staining for MMP9 **(b)** and SIRT3 **(c)**. **(d)** IHC staining quantitation for **(b,c)**. **(e)** Representative images of IHC staining for 8-oxo-dG. **(f)** Quantitation of 8-oxo-dG for **(e)**. **(g)** GSH/GSSG ratio. **(h)** Nrf2 activity. **(i)** NFκB p65 activity. **(j)** MRSA colony-forming units. **(k)** Honokiol plasma levels after honokiol hydrogel administration in 24 h n = 5. *, *P* < 0.05, vs. CTL/VEH group, *P* < 0.05, vs. STZ/VEH group; #, *P* < 0.05, vs. STZ/Hydrogel A group. Data were expressed as mean ± SEM.

The therapeutic effects of honokiol hydrogels were evaluated in diabetic wound models. Compared with non-diabetic controls (CTL/VEH), diabetic mice (STZ/VEH) showed increased mRNA expression of MMP9 and NFκB p65, along with reduced Nrf2 and SIRT3 expression (Figure 6a). These alterations were partially reversed by Hydrogel A and more effectively restored by Hydrogel B, except for NFκB p65. Consistent trends were observed at the protein level by IHC for MMP9 and SIRT3 (Figures 6b–d). Oxidative stress analysis revealed that diabetes increased 8-oxo-dG formation, reduced the GSH/GSSG ratio, and suppressed Nrf2 activity, all of which were partially rescued by Hydrogel A and more completely by Hydrogel B (Figures 6e–h). Similarly, diabetes-induced activation of NFκB p65 and elevated pro-inflammatory cytokine levels in wound tissues and plasma were attenuated by both hydrogels, with greater efficacy observed for Hydrogel B (Figure 6i, Supplementary Figures S3, S4). Microbial analysis showed that diabetic wounds exhibited a markedly higher MRSA burden compared to controls, which was significantly reduced by Hydrogel A and further decreased by Hydrogel B, approaching non-diabetic levels (Figure 6j). Pharmacokinetic analysis demonstrated that Hydrogel A produced a rapid peak in plasma honokiol followed by a decline, whereas Hydrogel B exhibited a slower onset but sustained systemic exposure, consistent with its prolonged-release profile (Figure 6k).

### Topical honokiol hydrogel accelerates diabetic wound healing

**FIGURE 7:**
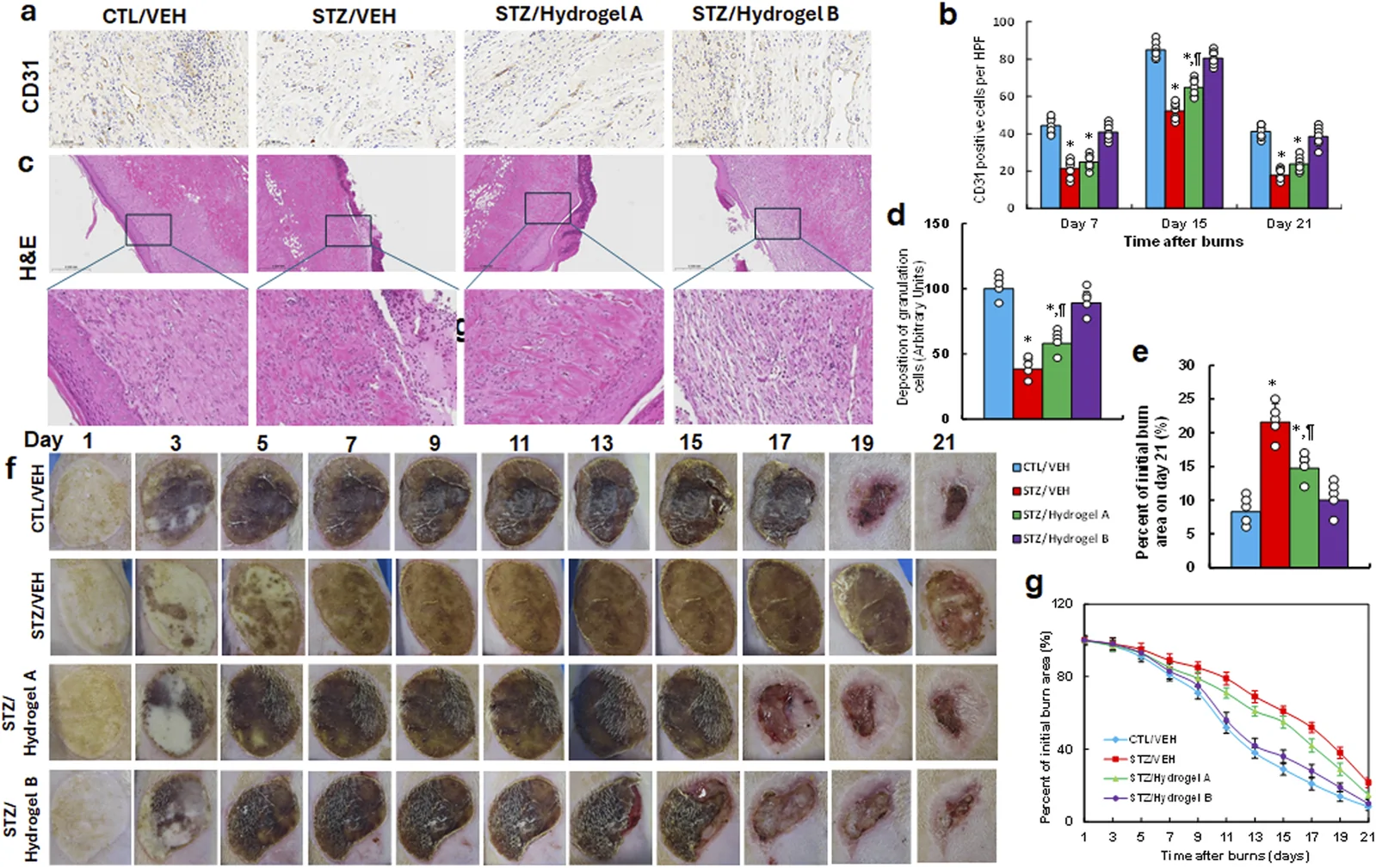
Topical administration of honokiol hydrogel accelerates diabetic wound healing. Mouse models with cutaneous burn injury received CTL or STZ treatment and then received topical administration of vehicle (VEH), Hydrogel A or Hydrogel B. The wounds were then isolated for analysis and the healing process was evaluated. **(a)** Representative IHC staining for CD31 on day 15 after burns. **(b)** Quantitative numbers of CD31 positive per HPF area on day 7, 15 and 21. **(c)** H&E staining of granulation cells on day 21 after burns. **(d)** Granulation cell deposition from **(c)**. **(e)** Quantitation of burn area on day 21. **(f)** Photographs of representative wounds from day 1–21 after burns. **(g)** Graphical depiction of wound areas on different days after burns. n = 7. *P* < 0.05, vs. CTL/VEH group, *P* < 0.05, vs. STZ/VEH group. Data were expressed as mean ± SEM.

The therapeutic efficacy of honokiol hydrogels in diabetic wound repair was evaluated *in vivo*. Compared with non-diabetic controls, diabetic mice exhibited significantly reduced CD31^+^ cell density at days 7, 15, and 21 post-burn, indicating impaired angiogenesis; this effect was partially restored by Hydrogel A and more completely by Hydrogel B (Figures 7a,b, Supplementary Figures S5a,b). Histological analysis by H&E staining at day 21 revealed reduced granulation tissue formation in diabetic wounds, which was partially rescued by Hydrogel A and more effectively restored by Hydrogel B (Figures 7c,d). Consistently, longitudinal wound monitoring demonstrated delayed healing in diabetic mice, whereas topical treatment with honokiol hydrogels significantly accelerated wound closure, with Hydrogel B showing the greatest improvement (Figures 7e–g).

### Schematic model of honokiol hydrogel–mediated enhancement of diabetic wound repair

**FIGURE 8:**
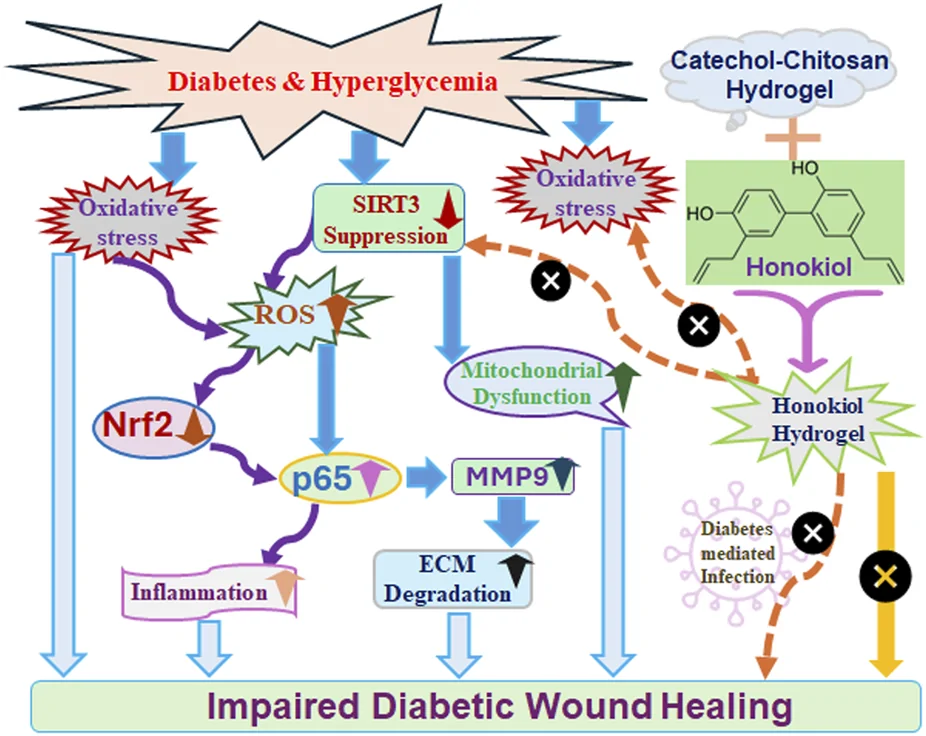
Schematic model for honokiol hydrogel-mediated acceleration of diabetic wound healing. Abbreviations: ECM: extracellular matrix; MMP9: Matrix Metalloproteinase-9; Nrf2: nuclear factor erythroid 2-related factor 2; p65: nuclear factor NFκB p65 subunit; ROS: reactive oxygen species; SIRT3: Sirtuin 3.

Hyperglycemia-induced oxidative stress suppresses SIRT3, leading to increased ROS production, Nrf2 inhibition, and NFκB p65 activation. These events promote MMP9-mediated ECM degradation, inflammation, and mitochondrial dysfunction, ultimately impairing wound healing. In contrast, honokiol hydrogel accelerates diabetic wound repair by restoring SIRT3 signaling, reducing oxidative stress and inflammation, and providing antimicrobial activity (Figure 8).

## Discussion

In this study, we demonstrate that honokiol reverses hyperglycemia-induced dysfunction by restoring SIRT3 signaling, thereby reducing oxidative stress, inflammation, mitochondrial damage, and ECM degradation. To translate this mechanism, we developed sprayable honokiol-loaded hydrogels with tunable crosslinking. Both formulations improved diabetic wound healing, with the densely crosslinked Hydrogel B showing superior efficacy due to greater stability and sustained release. Overall, these results connect honokiol-mediated mitochondrial protection with a practical biomaterial-based therapeutic strategy.

### Honokiol hydrogel formulation and functional characterization

Both moderate (Hydrogel A) and dense (Hydrogel B) crosslinked honokiol hydrogels were successfully fabricated using catechol-modified chitosan and oxidized dextran, forming homogeneous networks without phase separation (Lee et al., 2007). The two formulations exhibited distinct physicochemical properties associated with their crosslinking density. Hydrogel A showed favorable sprayability and moderate gelation, adhesion, and honokiol release, enabling rapid early bioactivity. In contrast, Hydrogel B displayed faster gelation, stronger tissue adhesion, greater structural stability at physiological temperature, and slower yet prolonged drug release (Ducheyne et al., 2012). Both hydrogels exhibited robust antioxidant and antimicrobial activities, although Hydrogel A produced stronger early-phase effects due to faster honokiol diffusion, whereas Hydrogel B maintained longer-lasting activity through sustained release (Qu et al., 2018). Cytocompatibility assays confirmed high viability of keratinocytes, fibroblasts, and endothelial cells for both formulations, with only minor reductions observed in the denser hydrogel. These results indicate that crosslinking density provides a practical strategy to tune hydrogel performance by balancing injectability, adhesion, and sustained therapeutic delivery.

### SIRT3–Nrf2–p65–MMP9 signaling axis in diabetic wound pathology

Diabetic wound healing is characterized by persistent oxidative stress, chronic inflammation, and dysregulated ECM remodeling (Long et al., 2016). Our results identify the SIRT3–Nrf2–p65–MMP9 signaling axis as a key molecular pathway connecting these pathological processes. Hyperglycemia markedly suppressed SIRT3 expression, resulting in impaired mitochondrial function and elevated accumulation of reactive oxygen species (ROS) (Yang et al., 2020). Elevated ROS impaired Nrf2-mediated antioxidant signaling while promoting activation of NFκB p65, a major inflammatory transcription factor. Increased p65 activity subsequently enhanced transcription of matrix-degrading enzymes such as MMP9, resulting in excessive ECM degradation and disruption of tissue architecture required for effective repair (Lobmann et al., 2002). These findings are consistent with accumulating evidence indicating that mitochondrial dysfunction and redox imbalance are central drivers of impaired wound healing in diabetes. By integrating upstream mitochondrial regulation (SIRT3), antioxidant signaling (Nrf2), inflammatory activation (p65), and ECM degradation (MMP9), our study provides a comprehensive framework linking metabolic stress to defective tissue repair (Zhang et al., 2023).

### Honokiol activation of the SIRT3–Nrf2 antioxidant network

Honokiol has been increasingly recognized as a bioactive natural compound with antioxidant, anti-inflammatory, and mitochondrial-protective properties. Consistent with previous reports, our findings indicate that honokiol exerts cytoprotective effects primarily through activation of the SIRT3-dependent antioxidant network. As an activator of the mitochondrial NAD^+^-dependent deacetylase SIRT3, honokiol promotes deacetylation of metabolic and antioxidant enzymes such as SOD2, thereby enhancing mitochondrial metabolism and reducing ROS accumulation. SIRT3 activation also facilitates stabilization of Nrf2 by alleviating oxidative stress and limiting its degradation through the Keap1–Cullin3 ubiquitin ligase complex (Kensler et al., 2007; Senyuk et al., 2021). In addition, honokiol may promote Nrf2 nuclear translocation through AMPK–PGC1α signaling pathways and potential electrophilic modification of Keap1 cysteine residues. Activation of this coordinated signaling network enhances transcription of antioxidant and detoxification genes including HO1, NQO1, and GPx, strengthening cellular redox defense. The resulting reduction in oxidative stress further suppresses NFκB–mediated inflammatory signaling and improves mitochondrial quality control. These mechanisms underscore the therapeutic relevance of targeting mitochondrial redox regulation in diabetic wound repair.

### Honokiol hydrogel modulates macrophage polarization and inflammatory responses

Macrophage polarization is a key determinant of inflammatory resolution and tissue regeneration during wound healing (Wynn and Vannella, 2016). Under diabetic conditions, hyperglycemia promotes a persistent pro-inflammatory M1-like macrophage phenotype, which contributes to chronic inflammation and impaired tissue repair. Consistent with this concept, our findings demonstrate that honokiol-loaded hydrogels effectively attenuated hyperglycemia-induced macrophage polarization in bone marrow–derived macrophages. Hyperglycemic stimulation markedly increased M1-associated markers, including inducible nitric oxide synthase (iNOS), NFκB p65 activation, and secretion of the pro-inflammatory cytokines TNFα and IL6, while reducing the M2-associated marker Arg1. Treatment with honokiol hydrogels reversed these alterations by suppressing M1 polarization and enhancing M2-associated responses (Li et al., 2021). Although IL10 and TGFβ were only modestly affected by hyperglycemia, likely because these cytokines are regulated by broader inflammation-resolution pathways rather than glucose-induced inflammatory signaling alone, honokiol hydrogels significantly enhanced their production, further supporting a shift toward a reparative macrophage phenotype. Notably, Hydrogel B exhibited greater immunomodulatory efficacy than Hydrogel A, likely due to its sustained release of honokiol. Immunostaining further confirmed these findings, showing diffuse cytoplasmic iNOS localization in M1-like macrophages and perinuclear enrichment of Arg1 in M2-like macrophages. Collectively, these results indicate that sustained honokiol delivery restores macrophage balance and mitigates hyperglycemia-driven inflammatory responses, thereby creating a more favorable immune microenvironment for diabetic wound healing.

### Hydrogel B as an optimized formulation for diabetic wound repair

Among the two formulations, Hydrogel B demonstrated the most favorable therapeutic profile for diabetic burn wound repair. Its denser crosslinked structure enables sustained honokiol release, resulting in prolonged antimicrobial and antioxidant activity. Continuous honokiol exposure is particularly beneficial in diabetic wounds characterized by persistent oxidative stress, as it supports sustained activation of antioxidant pathways such as Nrf2/HO-1, preserves mitochondrial function, and reduces lipid peroxidation (Long et al., 2016). In addition, Hydrogel B exhibits enhanced tissue adhesion and retention on irregular burn surfaces, facilitating stable local drug availability at the wound site (Lee and Konst, 2014). These properties contribute to improved angiogenesis, enhanced extracellular matrix remodeling, and accelerated tissue regeneration. Consistent with these advantages, *in vivo* experiments demonstrated significantly improved wound closure in the Hydrogel B-treated group compared with Hydrogel A. Although Hydrogel B has certain limitations, including slightly increased viscosity that may affect sprayability and minor reductions in cytocompatibility, these issues could be further optimized through formulation refinement. Notably, Hydrogel A provides faster honokiol release with stronger early antimicrobial and antioxidant effects, whereas Hydrogel B achieves more sustained drug delivery and superior long-term wound healing outcomes. Although a combined burst and sustained release strategy may provide additional therapeutic benefits, our findings indicate that sustained honokiol delivery alone is sufficient to promote effective wound repair and may represent a more suitable approach for clinical translation. Importantly, the plasma honokiol concentration following hydrogel application remained relatively low (approximately 100–500 ng/mL), indicating limited systemic exposure. Given that the local honokiol loading concentration within the hydrogel was approximately 1.0 mg/mL, which is substantially higher than the effective concentration used *in vitro* (5 μM, ∼1.3 μg/mL), the therapeutic effects are likely mediated predominantly through sustained local delivery at the wound site rather than systemic pharmacological activity. The limited systemic absorption may also contribute to the favorable safety profile of the honokiol hydrogel by minimizing potential off-target effects.

### Study limitations

Several limitations should be acknowledged. First, although our study identifies the SIRT3–Nrf2–p65–MMP9 signaling axis as a key mechanism underlying honokiol-mediated protection, genetic or pharmacological inhibition studies were not performed to definitively confirm pathway causality. Second, the diabetic burn wound model used in this study represents an acute experimental injury and may not fully capture the complexity of chronic diabetic ulcers observed clinically. Third, long-term biosafety, pharmacokinetics, and biodegradation profiles of the honokiol hydrogel require further investigation prior to clinical translation. Addressing these issues in future studies will help strengthen the mechanistic understanding and therapeutic applicability of this platform. Fourth, the murine dorsal wound model was established without a splinting ring. Therefore, wound closure likely reflected both wound contraction and tissue regeneration, and their individual contributions could not be quantitatively distinguished. Future studies using a splinted wound model are warranted. Fifth, the grafting efficiency and degree of catechol substitution were not quantitatively determined. Future studies incorporating detailed chemical characterization of catechol modification will provide further insight into the relationship between polymer modification degree and hydrogel performance. Finally, SIRT3 inhibition or knockdown experiments were not performed to directly confirm its causal role in honokiol-mediated protection. Although honokiol restored SIRT3 expression and improved therapeutic outcomes, these findings indicate an association rather than definitive causality. Future studies using SIRT3-specific inhibition or silencing are needed to further validate this mechanism.

### Conclusions and future perspectives

In summary, our study demonstrates that honokiol counteracts hyperglycemia-induced cellular injury by restoring SIRT3 expression and suppressing oxidative stress, inflammation, mitochondrial dysfunction, and ECM degradation. Mechanistically, honokiol activates the SIRT3-dependent antioxidant network, leading to recovery of Nrf2 signaling and inhibition of NFκB p65 activation and its downstream effector MMP9, thereby integrating mitochondrial regulation, redox balance, inflammatory signaling, and matrix remodeling in diabetic wound pathology. To translate these mechanistic findings into a therapeutic approach, we developed a sprayable catechol–chitosan honokiol hydrogel and demonstrated its efficacy in a diabetic burn mouse model. While both formulations exhibited antioxidant, antimicrobial, and cytocompatible properties, the densely crosslinked sustained-release formulation (Hydrogel B) achieved superior therapeutic outcomes, including improved redox homeostasis, enhanced mitochondrial function, reduced inflammatory responses and bacterial burden, enhanced angiogenesis and granulation tissue formation, thereby promoting faster wound closure. These findings suggest that sprayable honokiol hydrogels represent a promising multifunctional therapeutic platform for infection-resistant healing of diabetic burn wounds and highlight restoration of SIRT3-mediated mitochondrial signaling as a potential therapeutic strategy for improving tissue repair under diabetic conditions.

## Data Availability

The original contributions presented in the study are included in the article/Supplementary Material, further inquiries can be directed to the corresponding authors.
