# Supplementary material for: Targeting SIRT3 dysfunction with a sprayable honokiol hydrogel for diabetic burn wound repair
Source: Front Pharmacol. 2026 Sep 8;17:1912136. doi: 10.3389/fphar.2026.1912136 (PMC13593475; doi:10.3389/fphar.2026.1912136)
Supplement: Supplementary file 1 [file DataSheet1.pdf]

Targeting SIRT3 Dysfunction with a Sprayable Honokiol Hydrogel for Diabetic Burn Wound Repair

Supplementary Materials

Data S1: Materials and Methods

Reagents and Materials. Antibodies for iNOS (ab115819), MMP9 (ab73734), Nrf2 (ab137550) and SIRT3 (ab217319) were purchased from Abcam. Antibodies for NFκB p65 (#8242) and Histone H3 (#9715) were obtained from Cell Signaling Technology. Antibody for β-actin (sc-47778) was obtained from Santa Cruz Biotechnology. The 8-oxo-dG antibody (4354-MC-050) was obtained from Novus Biologicals. Protein concentration was measured using the Coomassie Protein Assay Kit (Pierce Biotechnology). The NE-PER Nuclear and Cytoplasmic Extraction Kit (#78835) was purchased from Thermo Scientific, China. NFκB p65 activity was measured by TransAM® NFκB p65 Chemi Kit (#40597, from Active Motif) per manufacturers' instructions.
Catechol-modified chitosan (2% w/v, 150-200 kDa, >90% deacetylated) was prepared from medical-grade chitosan (Qingdao Yunzhou Biochemistry Co., Ltd, China) by dopamine conjugation, and oxidized dextran (70 kDa) was generated by sodium periodate oxidation of dextran (Sigma, China). Honokiol (≥98% purity, Sigma) was used as the bioactive compound, and formulation solvents including propylene glycol, medium-chain triglycerides (MCT oil), and dimethyl sulfoxide (DMSO) were purchased from Sigma, China.
Cell culture. HaCaT human keratinocytes, NIH 3T3 mouse fibroblasts and human umbilical vein endothelial cells (HUVEC) were obtained from Procell Life Science & Technology Co., Ltd. (Wuhan, China). Bone marrow–derived macrophages (BMMs) were isolated from C57BL/6 mice and differentiated in vitro using recombinant mouse M-CSF purchased from Sino Biological (Beijing, China). All cells were cultured in Dulbecco's Modified Eagle Medium supplemented with 10% fetal bovine serum (FBS) at 37°C in a humidified incubator with 5% CO2.
Bacteria. Methicillin-resistant *Staphylococcus aureus* (MRSA, ATCC 43300), *Pseudomonas aeruginosa* (PA, ATCC 27853), and *Escherichia coli* (Ecoli, ATCC 25922), were obtained from China Center of Industrial Culture Collection (CICC, China).

Hydrogel Preparation. Catechol-modified chitosan (2% w/v, degree of deacetylation >90%, MW 150–200 kDa) was dissolved in 0.1M acetic acid under magnetic stirring (300 rpm) for 2 h at room temperature until clear and homogeneous; the pH was then adjusted to 6.5 with 1 M NaOH while continuously monitoring with a calibrated pH meter to avoid local precipitation. Oxidized dextran (MW ~70 kDa; ~30% oxidation) was prepared by reacting 10.0 g dextran in 100.0 mL deionized water with 1.50 g sodium periodate in the dark at 25 °C for 2 h, quenching with 2.00 mL ethylene glycol, dialyzing (MWCO 3.5 kDa) against deionized water for 48 h (water changes every 6 h), and lyophilizing to yield dry oxidized dextran. Honokiol (≥98% purity, Sigma-Aldrich) was prepared as a 100 mg/mL stock in DMSO (to limit added solvent volume) and added dropwise to the catechol-chitosan solution under gentle stirring (100rpm) to give a final honokiol concentration of 1.0 mg/mL (0.10% w/v) in the catechol-chitosan/honokiol mixture (for each 10.0 mL final hydrogel this corresponds to 10.0 mg honokiol; this addition uses 0.100 mL of 100 mg/mL stock, producing 1.0% v/v DMSO in the final formulation). Two crosslinking densities were prepared by adding oxidized dextran to 10.0 mL of the catechol-chitosan/honokiol solution: Hydrogel A (moderate crosslinking) = 120 mg oxidized dextran per 10.0 mL; Hydrogel B (dense crosslinking) = 180 mg oxidized dextran per 10.0 mL; control hydrogels were prepared identically but without honokiol. Gelation was initiated at room temperature (22–25°C) under sterile conditions by combining catechol-chitosan/honokiol with oxidized dextran and vortexing gently for 10 s; gel formation was confirmed by vial inversion and rheology (G' > G'', 25 °C, 0.1–10 Hz, 1% strain). Hydrogels were

homogeneous with no visible phase separation and were stored at 4 °C under aseptic conditions until use. The 1.0 mg/mL choice balances reported in vivo/biomaterial studies showing honokiol efficacy in hydrogel wound dressings and human dermatology patch testing that found 1% (10 mg/mL) topical formulations non-irritating (thus 1.0 mg/mL is conservative and likely safe) while keeping DMSO content low (≈1% v/v) (1, 2).

Sprayability. To evaluate sprayability, 5 mL of hydrogel was loaded into sterile syringes fitted with 25G needles. The hydrogel was sprayed onto sterile Petri dishes placed 10 cm away. Extrusion force was quantified by pressing the syringe plunger against an analytical balance and recording the maximum applied force required to initiate and sustain flow. Consistent spray patterns and manageable extrusion force were used as indicators of suitability for topical delivery.

Gelation. Gelation time was determined using the vial inversion method. Aliquots (1 mL) of freshly mixed hydrogel were placed into sterile glass vials at room temperature. The vials were inverted at 10-s intervals, and gelation was confirmed when no visible flow was observed within 1 min. The reproducibility of gelation was confirmed in triplicate for each preparation.

Adhesion. Adhesive strength was quantified using ex vivo porcine dermis as a tissue substrate. Strips of dermis (1 × 2 cm) were cleaned and gently blotted dry. A 100 µL aliquot of hydrogel was applied between two dermis strips, which were then pressed together under a constant weight of 200 g for 2 min to allow bonding. The bonded samples were subsequently subjected to incremental weights until detachment occurred, and the maximum weight sustained before separation was recorded as the detachment force.

Stability. Hydrogel formulations were aliquoted into sterile tubes and stored at either 4 °C or 37 °C for up to 14 days. Samples were visually inspected on days 0, 7, and 14 for phase separation, turbidity, or color change. Stability was further assessed by measuring OD600 of hydrogel extracts in PBS to monitor aggregation or degradation over time. A formulation was considered stable if no phase separation and less than 10% change in OD600 were observed over the storage period.

Drug Release. Drug release kinetics were determined using the dialysis bag method. Hydrogel samples (equivalent to 1 mg honokiol) were sealed in dialysis bags with a 3.5 kDa molecular weight cut-off and immersed in 10 mL PBS (pH 7.4) at 37 °C with constant shaking (100 rpm). At predetermined time points (0.5, 1, 2, 4, 8, 24, and 48 h), 1 mL aliquots were withdrawn and replaced with fresh PBS. Honokiol concentration in the aliquots was quantified by UV–Vis spectroscopy at 290 nm using a standard calibration curve.

Antioxidant Activity. The antioxidant activity of hydrogel extracts was assessed using a DPPH radical scavenging assay. Briefly, 100 µL of hydrogel extract was mixed with 900 µL of freshly prepared 0.1 mM DPPH solution in methanol, vortexed, and incubated for 30 min in the dark at room temperature. Absorbance at 517 nm was recorded using a microplate reader, and the percentage scavenging activity was calculated as [(Acontrol – Asample)/Acontrol] × 100, where Acontrol represented the absorbance of the DPPH solution alone.

Antimicrobial Activity by Agar Diffusion. For agar diffusion assays, MRSA, *Pseudomonas aeruginosa*, and *Escherichia coli* cultures were grown in LB broth to mid-log phase and adjusted to 10^6 CFU/mL. LB agar plates were uniformly inoculated with each bacterial suspension, and sterile filter paper discs were loaded with 50 µL of hydrogel before being placed on the agar surface. Plates were incubated at 37 °C for 24 h, after which inhibition zones were measured in millimeters using a digital caliper.

Antimicrobial Activity by Minimum Inhibitory Concentration (MIC)21. MIC values were determined by broth microdilution. Hydrogel extracts were serially diluted two-fold in LB broth in 96-well plates to generate concentration gradients. Bacterial suspensions of MRSA, *P. aeruginosa*, and *E. coli* were adjusted to 10^5 CFU/mL and added to each well. Plates were incubated at 37°C for 24 h, and bacterial growth was assessed by measuring OD@600nm. The

MIC was defined as the lowest concentration at which growth inhibition resulted in OD@600nm ≤ 0.05 above background.

Cytocompatibility. The cytocompatibility of hydrogel extracts was evaluated in HaCaT keratinocytes, NIH 3T3 fibroblasts, and HUVECs. Cells were seeded in 96-well plates at a density of 5×10^3 cells/well and allowed to adhere overnight. Hydrogel extracts were added to the wells and incubated for 24 h under standard culture conditions (37°C, 5% CO_2). Cell viability was assessed using the MTT assay by adding 10 µl of 5 mg/mL MTT solution to each well and incubating for 4 h. The resulting formazan crystals were dissolved in DMSO, and absorbance was measured at 570 nm. Cell viability ≥80% relative to untreated controls was considered biocompatible.

RT reaction and real-time quantitative PCR. Total RNA from treated cells was extracted using the RNeasy Micro Kit (Qiagen), and RNA was reverse transcribed using the Omniscript RT Kit (Qiagen). Primers were designed using Primer3Plus software with a melting temperature (Tm) of 60°C, a primer length of 21 bp, and an expected amplicon size of 140–160 bp (Table S1). Primer specificity was confirmed by agarose gel electrophoresis, and amplification efficiencies ranged from 1.9 to 2.1. Real-time quantitative PCR was performed on an iCycler iQ system (Bio-Rad) using the QuantiTect SYBR Green PCR Kit (Qiagen). The PCR conditions consisted of an initial denaturation at 95°C for 8 min, followed by 45 cycles of denaturation at 95°C and annealing/extension at 60°C and 72°C for 10 s each. One microliter of cDNA was used in each PCR reaction. β-Actin was used as the internal reference gene. Relative mRNA expression was calculated using the comparative threshold cycle (2^-ΔΔCt) method, where ΔCt = Ct(target gene) − Ct(β-actin), ΔΔCt = ΔCt(experimental) − ΔCt(reference), and fold change = 2^-ΔΔCt. For graphical presentation in figures, the mean relative expression level of the control group was set to 100 arbitrary units, and all other groups were expressed relative to this normalized control value.

Western blotting. Cells were lysed in an ice-cold lysis buffer (0.137M NaCl, 2mM EDTA, 10% glycerol, 1% NP-40, 20mM Tris base, pH 8.0) with protease inhibitor cocktail (Sigma). The proteins were separated in 10% SDS-PAGE and transferred to the PVDF membrane, which was then blotted using primary antibodies (1:1000) and then simultaneously incubated with the differentially labeled species-specific secondary antibodies, anti-RABBIT IRDye™ 800CW (green) and anti-MOUSE (or goat) ALEXA680 (red). Membranes were scanned and quantitated using the ODYSSEY Infrared Imaging System (LI-COR, NE) (3).

MTT assay. Approximately 0.5×10^6 cells were seeded into 12-well plates and exposed to the indicated treatments upon reaching ~80% confluence. Cellular metabolic activity was assessed using the MTT (3-(4,5-dimethylthiazol-2-yl)-2,5-diphenyltetrazolium bromide) reduction assay. Briefly, cells were washed with PBS and incubated with 0.2 mL of 0.3 mg/mL MTT solution at 25°C for 3 h. The resulting formazan crystals were dissolved overnight at 37°C in 0.1 mL of 10% SDS prepared in 0.01 M HCl. Absorbance was measured at 560 nm with background subtraction at 670 nm. The corrected absorbance values were normalized to the corresponding cell number and expressed as OD per 10^6 cells, representing cellular metabolic activity on a per-cell basis rather than total viable cell number (4).

Measurement of oxidative stress. Intracellular reactive oxygen species (ROS) levels were quantified using CM-H_2DCFDA (10µM; Invitrogen) following incubation at 37°C for 45 min, and fluorescence intensity was measured at 485/530 nm and expressed in arbitrary units. The formation of 8-oxo-2'-deoxyguanosine (8-oxo-dG) was assessed by immunostaining and quantified using ImageJ software. The intracellular GSH/GSSG ratio was measured using the GSH/GSSG-Glo™ Assay Kit (Promega, #V6611) according to the manufacturer's instructions.

Nrf2 transcriptional activity was measured using the Nrf2 Transcription Factor Assay Kit (Colorimetric; Abcam, #ab207223). Absorbance values were normalized to the control group (set as 100%) and presented as relative Nrf2 DNA-binding activity. Methylglyoxal (MG) levels were quantified using the Methylglyoxal Assay Kit (Abcam, #ab241006), calculated from the manufacturer's standard curve, and presented as concentrations (5).

Immunostaining. Cells were cultured directly on 0.1% gelatin-coated coverslips placed in culture plates and subjected to the indicated treatments. For mitochondrial staining, cells were incubated with MitoTracker™ Red CMXRos (Thermo Fisher Scientific) at a final concentration of 100 nM in prewarmed culture medium for 30 min at 37°C, protected from light, followed by one wash with PBS. Cells were then fixed with 3.7% formaldehyde at 37°C for 15 min, permeabilized with 1% BSA + 0.2% Triton X-100 in PBS for 1 hour, and incubated with 40 μg/mL (1:50 dilution) of primary antibodies against iNOS, SIRT3, or 8-oxo-dG for 2 hours at room temperature. After three washes with PBS, FITC- or Texas Red-conjugated anti-mouse/rabbit secondary antibodies (1:100) were added and incubated for 1 hour. Following thorough washing, nuclei were counterstained with 4′,6-diamidino-2-phenylindole dihydrochloride (DAPI; #D9542, Sigma) to visualize nuclei. Fluorescence images were acquired using identical microscope settings for all samples, and fluorescence intensity was quantified using ImageJ software (6).

Measurement of mitochondrial function.
Mitochondrial DNA copies. The genomic DNA was extracted from cells using a QIAamp DNA Mini Kit (Qiagen) and the mitochondrial DNA was extracted using the REPLI-g Mitochondrial DNA Kit (Qiagen). The purified DNA was used for the analysis of genomic β-actin (marker of the nuclear gene) and ATP6 (ATP synthase F0 subunit 6, marker of the mitochondrial gene) respectively using the qPCR method mentioned above. The primers for genomic β-actin: forward 5'-acc aca gct gag agg gaa atc -3' and reverse 5'- att gcc gat agt gat gac ctg-3'. The primers for ATP6: forward 5'- tag ggc ttc ttc ccc ata cat -3' and reverse 5'- tta gtg aga tgg ggg ttc ctt-3'. The mitochondrial DNA copies were obtained from relative ATP6 copies that were normalized by β-actin copies using the ΔΔCT method (7-9).
Intracellular ATP level. The intracellular ATP level was determined using the luciferin/luciferase-induced bioluminescence system. An ATP standard curve was generated at concentrations of 10^{-12}-10^{-3}M. Intracellular ATP levels were calculated and expressed as nmol/mg protein (7-9).
Mitochondria membrane potential. Mitochondrial membrane potential (MMP, Δψm) was measured using TMRE (from Molecular Probes T-669) staining. A 600 μM T-669 stock solution was prepared using DMSO. Cells were grown on coverslips and immersed in 600 nM TMRE for 20 min at 37°C to load them with dye. The labeling medium was then aspirated and the cells were immersed in 150 nM TMRE to maintain the equilibrium distribution of the fluorophore. The coverslips were mounted with live cells onto confocal microscopes to image the cells using 548 nm excitation/573 nm emission filters. The intensity of TMRE fluorescence was measured using Image J software. Data from 10-20 cells were collected for each experimental condition and mean values of fluorescence intensity ± SEM were calculated (5, 9, 10).
Caspase-3 activity was determined by the ApoAlert caspase assay kit (Clontech). Treated cells were harvested and 50 μg of proteins were incubated with the fluorogenic peptide substrate Ac-DEVD-7-amino-4-trifluoromethyl coumarin (AFC). The initial rate of free AFC release was measured using a FLx800 microplate reader (Bio-Tek) at excitation/emission wavelengths of 380/505nm, and enzyme activity was calculated as pmol/min/mg (8).

In vivo mouse experiments. Animal procedures adhered to the National Institutes of Health guidelines for the care and use of laboratory animals and received prior approval from the Institutional Animal Care and Use Committee (IACUC) at Tongren Hospital, Wuhan University. Animals were housed in a controlled facility with stable temperature and humidity, maintained on

a 12-hour light/dark cycle, and provided food and water ad libitum. All surgical/wounding procedures were performed under inhalational anesthesia and animals will receive peri- and post-operative analgesia per IACUC-approved veterinary recommendations. All end-point tissue collection will follow AVMA-approved euthanasia methods.

1. Diabetic Mouse Model (STZ-induced). C57BL/6 mice (8–10 weeks old) will be acclimated for at least 7 days before experiments and housed 3–5 per cage under a 12:12-h light-dark cycle with food and water ad libitum. Diabetes will be induced by intraperitoneal injection of streptozotocin (STZ, 50 mg/kg) once daily for 5 consecutive days after a 6–8 hour fast, with fresh STZ prepared in 0.05 M citrate buffer (pH 4.5–5.5) immediately before use. Control mice will receive citrate buffer injections. Non-fasting blood glucose will be measured 7 and 14 days after the final injection, and mice with glucose >250 mg/dL on two consecutive measurements will be considered diabetic and included in the study.

2. Wound Model. Mice will be anesthetized with isoflurane (3–4% induction, 1–2% maintenance) and receive analgesia per IACUC guidelines. The dorsal skin will be shaved and disinfected, and a 2-cm diameter full-thickness burn will be created using a heated copper pillar at 75°C for 15 seconds. To model infection, a subset of wounds will be inoculated immediately with 10^6 CFU MRSA in 10 µL PBS. Animals will then be randomized into four groups (n = 8 per group): non-diabetic control (CTL/VEH), diabetic untreated (STZ/VEH), diabetic + Hydrogel A (STZ/Hydrogel A), and diabetic + Hydrogel B (STZ/Hydrogel B).

3. Treatment. Hydrogels will be applied directly to the wound surface at 50 µL per wound under sterile conditions and covered with Tegaderm film dressing. Treatments will be administered once daily for 21 days, with the same volume of vehicle hydrogel applied to CTL/VEH and STZ/VEH groups. Wounds will be monitored daily for local irritation, infection progression, or adverse effects, and dressing changes will coincide with hydrogel reapplication.

4. Wound Healing Measurements. Digital photographs of wounds were taken every two days for 21 days, and wound area was quantified as a percentage of the original wound size using ImageJ software. At designated time points, wounds were excised and either snap-frozen or processed for histological analyses, including H&E staining and immunohistochemistry (IHC). Images were captured using a Carl Zeiss MIRAX MIDI slide scanner, and granulation tissue deposition was quantified with 3DHISTECH Pannoramic Viewer. Neovascularization was evaluated by immunohistochemical staining for CD31 on frozen tissue sections. Representative images were acquired at 200× magnification, and CD31-positive cells were quantified in five randomly selected high-power fields (HPFs) per tissue section by a blinded investigator. The average number of CD31-positive cells per HPF was used for statistical analysis. Cytokine levels, including TNFα, IL6, IL10, and TGFβ, were measured in wound tissues and plasma using ELISA kits (R&D Systems). Plasma concentrations of honokiol following topical application were determined by LC-MS/MS. Gene expression of MMP9, NFκB p65, Nrf2, and SIRT3 was analyzed from wound tissues, while oxidative stress was evaluated by measuring Nrf2 activity, the GSH/GSSG ratio, and 8-oxo-dG formation. Antimicrobial efficacy was determined by homogenizing wound tissues, plating serial dilutions on mannitol salt agar, and counting MRSA colony-forming units (CFUs) after 24 hours of incubation at 37°C. Pharmacokinetic profiling was performed in a separate cohort of mice (n = 5 per group), with plasma samples collected at 0.5, 1, 2, 6, 12, and 24 hours after hydrogel application. Honokiol was extracted using acetonitrile and quantified against standard calibration curves by LC–MS/MS (9, 11).

Preparation of bone marrow–derived macrophages (BMMs). BMMs were prepared by flushing femurs and tibiae of adult C57BL/6 mice with RPMI-1640, filtering through a 70-µm

strainer, lysing red blood cells, and culturing cells at 1.0-1.5×10^6 cells/mL in complete medium (RPMI-1640 supplemented with 10% FBS and 1% penicillin/streptomycin) containing recombinant M-CSF (20–30 ng/mL) for 7 days, with half-medium changes on day 3. Adherent BMDMs were harvested on day 7 by gentle scraping and plated at equal density for polarization under four conditions: untreated control (M0), M1 (LPS 100 ng/mL + IFN-γ 20 ng/mL), M2 (IL-4 20 ng/mL), or treatment plus polarizing stimulus for 18–24 h. Culture supernatants were collected for multiplex ELISA to quantify TNFα, IL6, IL10, and TGFβ levels. For assessment of NFκB activation, cells were harvested 30–60 min post-stimulation, lysed in RIPA buffer with protease and phosphatase inhibitors, and analyzed by SDS-PAGE and immunoblotting using antibodies against phospho-p65, total p65, and β-actin, with densitometric quantification normalized to total protein. These analyses enabled evaluation of macrophage polarization and inflammatory signaling in response to the different treatments.

Measurement of plasma honokiol. Plasma pharmacokinetics of honokiol were evaluated in a separate cohort of mice (n = 5 per group) by collecting blood samples at 0.5, 1, 2, 6, 12, and 24 h after topical hydrogel application, followed by centrifugation at 3,000 × g for 10 min to isolate plasma. Honokiol was extracted from 50 µL plasma using protein precipitation with three volumes of ice-cold acetonitrile containing an internal standard, vortex-mixed for 1 min, and centrifuged at 12,000 × g for 15 min at 4 °C. The supernatants were transferred to autosampler vials, evaporated under a gentle nitrogen stream, and reconstituted in 100 µL of 50% methanol prior to analysis. Quantification was performed using LC–MS/MS equipped with a C18 reverse-phase column, operated under a gradient of water with 0.1% formic acid (mobile phase A) and acetonitrile (mobile phase B), with honokiol detected in negative electrospray ionization mode using multiple reaction monitoring (MRM). Standard calibration curves were prepared by spiking blank mouse plasma with known concentrations of honokiol (10–5,000 ng/mL), and the lower limit of quantification (LLOQ) was set at 10 ng/mL. Plasma concentrations of honokiol were calculated by comparing sample peak areas to calibration curves and plotted to generate concentration–time profiles.

Immunohistochemistry (IHC). Wound tissues were sectioned into 10µm slices using a cryostat microtome. Sections were fixed in 2% paraformaldehyde for 15 minutes at room temperature, followed by post-fixation in methanol for 10 minutes at 4°C. After fixation, tissue sections were permeabilized and blocked with 1% bovine serum albumin (BSA) and 0.2% Triton X-100 in phosphate-buffered saline (PBS) for 1 hour at room temperature. Sections were then incubated with a primary antibody (40 µg/mL) for MMP9, SIRT3 or 8-oxo-dG for 2 hours at room temperature. After washing, samples were incubated with a horseradish peroxidase (HRP)-conjugated secondary antibody for 1 hour, and immunoreactivity was visualized using a 3,3'-diaminobenzidine (DAB) substrate solution until brown staining appeared. Sections were counterstained lightly with hematoxylin, dehydrated, and mounted with neutral mounting medium. Images were captured using a bright-field microscope, and the expression was quantified in 60 cells per group using ImageJ software (9).

Analysis of cytokines by ELISA. Cytokine levels were quantified using commercially available ELISA kits (TNFα, IL6, IL10, and TGFβ) from R&D Systems (China) following the manufacturer's instructions. Briefly, culture supernatants were collected 18-24 h after stimulation, centrifuged at 300×g for 5 min to remove debris, and stored on ice or at −80°C until analysis. Standards were prepared by serial dilution to generate a standard curve, and samples were diluted as needed to fall within the linear range. Wells of pre-coated 96-well plates were blocked for 1h at room temperature, then 100µL of standards or samples were added in duplicate and incubated 2h at room temperature. After washing, biotinylated detection antibody was applied for 1–2h, followed by HRP-conjugated streptavidin for 30-45 min. TMB substrate was added for color development,

and the reaction was stopped with acid solution. Absorbance was measured at 450 nm, and cytokine concentrations were calculated from the standard curve and expressed as pg/mL.

Statistical analysis. All data are presented as mean ± standard error of the mean (SEM). Normality was assessed using the Shapiro–Wilk test (12). To avoid pseudo-replication, the litter was used as the experimental unit where applicable. Statistical analyses were performed using SPSS version 22. Differences among multiple groups were analyzed by one-way ANOVA followed by Tukey's multiple comparisons test. A $P < 0.05$ was considered statistically significant.

REFERENCES

1. Wei Q, Jin Z, Zhang W, Zhao Y, Wang Y, Wei Y, et al. Honokiol@PF127 crosslinked hyaluronate-based hydrogel for promoting wound healing by regulating macrophage polarization. *Carbohydr Polym.* 2023;303:120469.
2. Li Y, Liang C, and Zhou X. The application prospects of honokiol in dermatology. *Dermatol Ther.* 2022;35(8):e15658.
3. Ceradini DJ, Yao D, Grogan RH, Callaghan MJ, Edelstein D, Brownlee M, et al. Decreasing intracellular superoxide corrects defective ischemia-induced new vessel formation in diabetic mice. *J Biol Chem.* 2008;283(16):10930-8.
4. Yao D, Li H, Gou Y, Zhang H, Vlessidis AG, Zhou H, et al. Betulinic acid-mediated inhibitory effect on hepatitis B virus by suppression of manganese superoxide dismutase expression. *Febs j.* 2009;276(9):2599-614.
5. Zhang H, Li L, Li M, Huang X, Xie W, Xiang W, et al. Combination of betulinic acid and chidamide inhibits acute myeloid leukemia by suppression of the HIF1alpha pathway and generation of reactive oxygen species. *Oncotarget.* 2017;8(55):94743-58.
6. Wang X, Lu J, Xie W, Lu X, Liang Y, Li M, et al. Maternal diabetes induces autism-like behavior by hyperglycemia-mediated persistent oxidative stress and suppression of superoxide dismutase 2. *Proc Natl Acad Sci U S A.* 2019;116(47):23743-52.
7. Zou Y, Lu Q, Zheng D, Chu Z, Liu Z, Chen H, et al. Prenatal levonorgestrel exposure induces autism-like behavior in offspring through ERbeta suppression in the amygdala. *Mol Autism.* 2017;8:46.
8. Yao D, Shi W, Gou Y, Zhou X, Yee Aw T, Zhou Y, et al. Fatty acid-mediated intracellular iron translocation: a synergistic mechanism of oxidative injury. *Free Radic Biol Med.* 2005;39(10):1385-98.
9. Zhang H, Li L, Chen Q, Li M, Feng J, Sun Y, et al. PGC1beta regulates multiple myeloma tumor growth through LDHA-mediated glycolytic metabolism. *Mol Oncol.* 2018;12(9):1579-95.
10. Kong D, Zhan Y, Liu Z, Ding T, Li M, Yu H, et al. SIRT1-mediated ERbeta suppression in the endothelium contributes to vascular aging. *Aging Cell.* 2016.
11. Thangarajah H, Yao D, Chang EI, Shi Y, Jazayeri L, Vial IN, et al. The molecular basis for impaired hypoxia-induced VEGF expression in diabetic tissues. *Proc Natl Acad Sci U S A.* 2009;106(32):13505-10.
12. Ramezani G, Norouzi A, Arabshahi SKS, Sohrabi Z, Zazoli AZ, Saravani S, et al. Study of medical students' learning approaches and their association with academic performance and problem-solving styles. *J Educ Health Promot.* 2022;11:252.

Table S1. Sequences of primers for the real time quantitative PCR (qPCR)

Gene	Species	Analysis	Forward primer (5'→3')	Reverse primer (5'→3')
β-actin	Human	mRNA	gatgcagaaggagatcactgc	atactcctgcttgctgatcca
MMP9	Human	mRNA	gtcttccccttcactttcctg	gccacgaggaacaaactgtat
Nrf2	Human	mRNA	agatagtgcccctggaagtgt	caggcaattctttctctggtg
SIRT3	Human	mRNA	gaaactacaagcccaacgtca	caaaggttccatgagcttcaa
NFκB p65	Human	mRNA	agcttgtaggaaaggactgcc	ggttgttgttggtctggatgc
β-actin	Mouse	mRNA	tcttgggtatggaatcctgtg	atctccttctgcatcctgtca
MMP9	Mouse	mRNA	atttcttcaaggacggttggt	aagaaaaccctcttggtctgc
Nrf2	Mouse	mRNA	ctcctcgctggaaaaagaagt	aattcatcgccaaaatctgtg
SIRT3	Mouse	mRNA	aacccgacattgtgttctttg	gggcactgatttctgtactgc
iNOS	Mouse	mRNA	catgctaatgcgaaaggtcat	ttgccttatactggtccatgc
NFκB p65	Mouse	mRNA	cctcatctttccctcagagcc	tggtatctgtgcttctctcgc
Arg1	Mouse	mRNA	acagggctcctttcaggacta	agtcagtccctggcttatggt

FIGURE S1

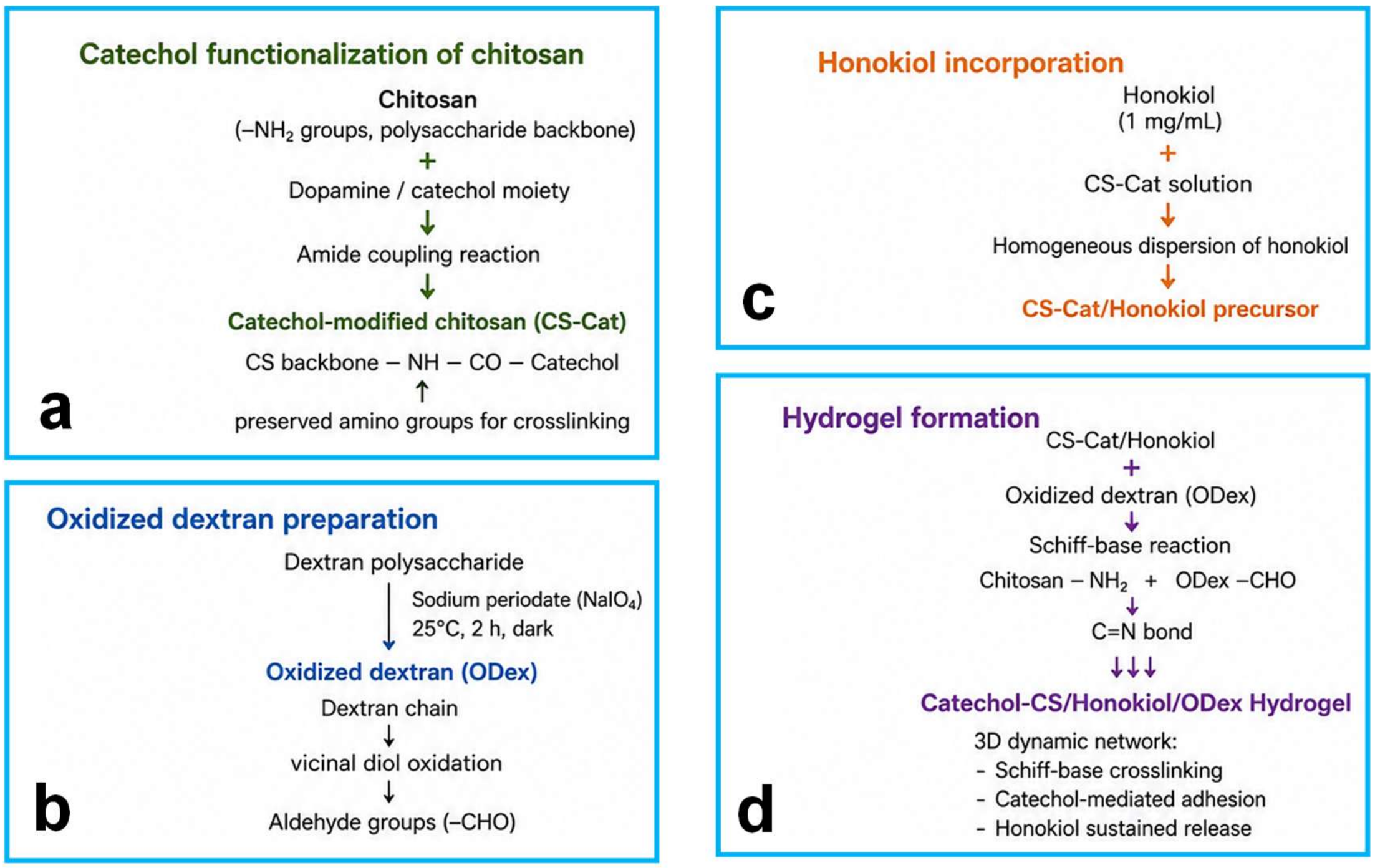

Figure S1. Schematic illustration of hydrogel synthesis. (a) catechol functionalization of chitosan. (b) oxidized dextran preparation. (c) honokiol incorporation. (d) hydrogel formation.

FIGURE S2

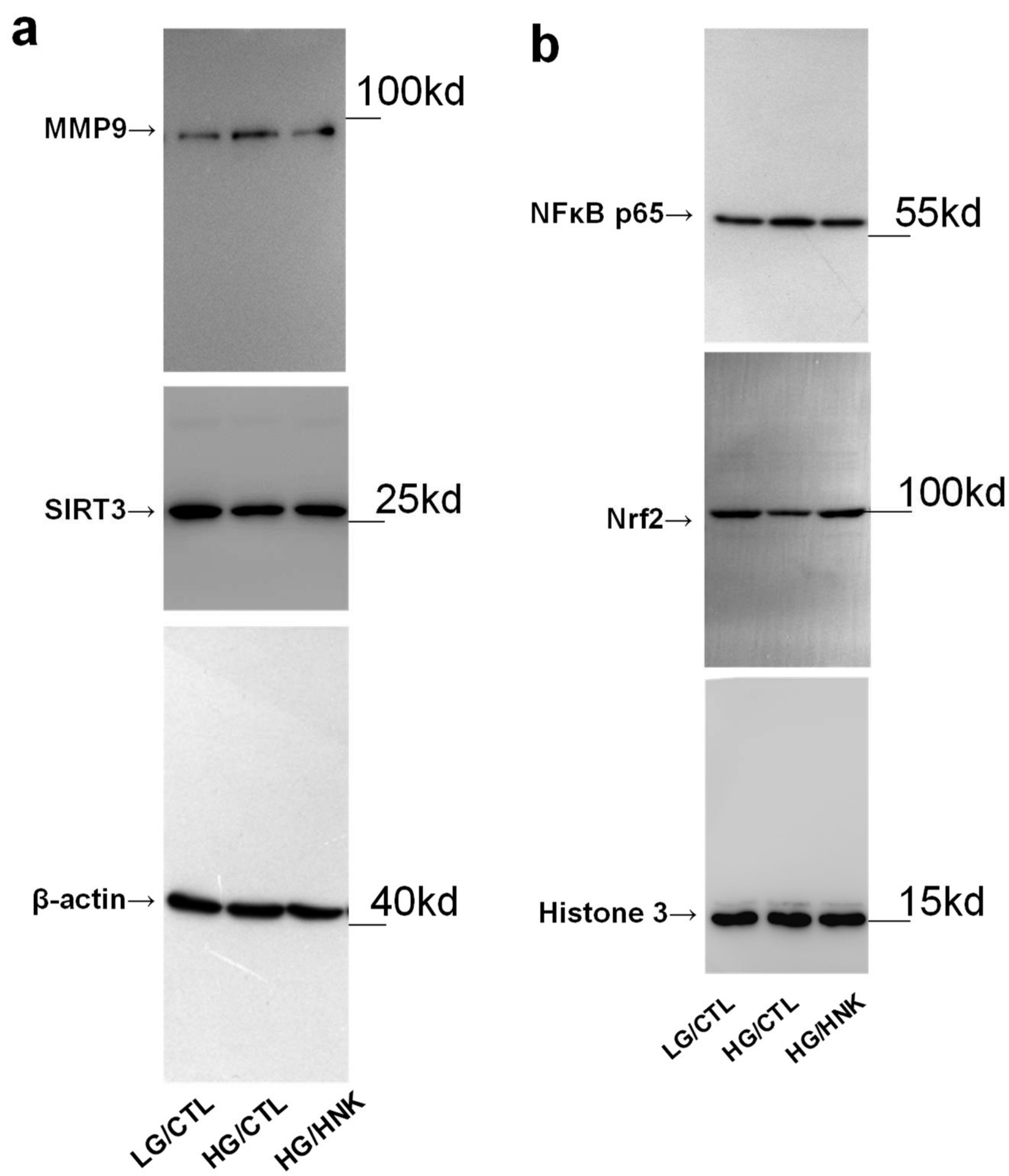

Figure S2. Representative pictures of full blots for Western Blotting. (a) Full blots for Figure 1c. (b) Full blots for Figure 1d.

FIGURE S3

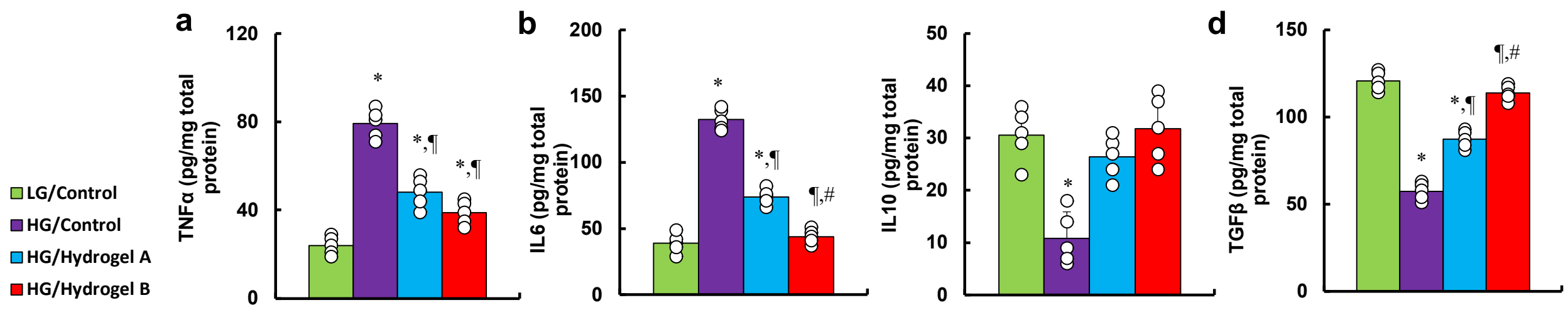

Figure S3. Honokiol hydrogel administration ameliorates diabetes-mediated pro-inflammatory cytokine release in wound tissues. Mouse models with cutaneous burn injury received CTL or STZ treatment and then received topical administration of vehicle (VEH), Hydrogel A or Hydrogel B, and the wound tissues were then isolated on day 7 after burns for assay of pro-inflammation cytokines for TNFα (a), IL6 (b), IL10 (c) and TGFβ (d). n=5. *, $P<0.05$, vs LG/CTL group; ¶, $P<0.05$, vs HG/CTL group. Data were expressed as mean ± SD.

FIGURE S4

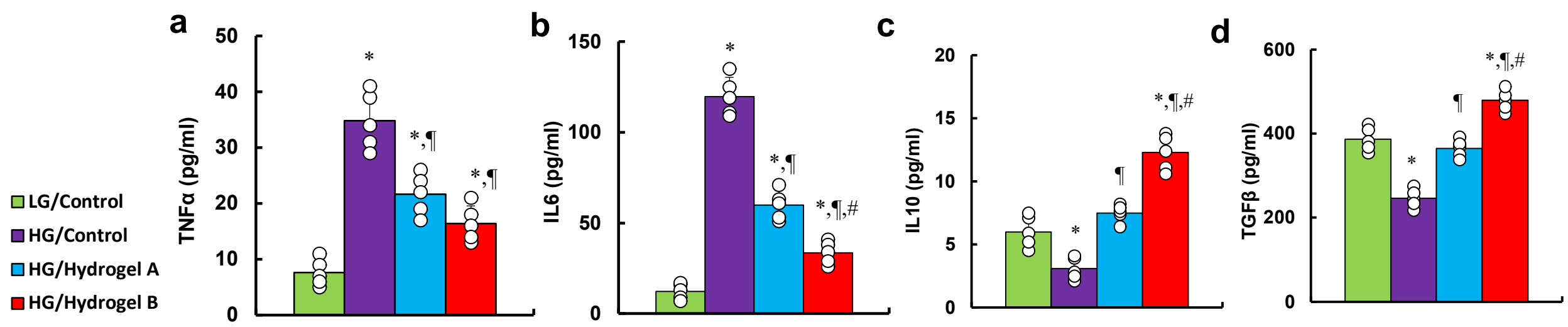

Figure S4. Honokiol hydrogel administration ameliorates diabetes-mediated pro-inflammatory cytokine release in wound tissues. Mouse models with cutaneous burn injury received CTL or STZ treatment and then received topical administration of vehicle (VEH), Hydrogel A or Hydrogel B, and the plasma was then isolated on day 7 after burns for assay of pro-inflammation cytokines for TNFα (a), IL6 (b), IL10 (c) and TGFβ (d). n=5. *, P<0.05, vs LG/CTL group; ¶, P<0.05, vs HG/CTL group. Data were expressed as mean ± SD.

FIGURE S5

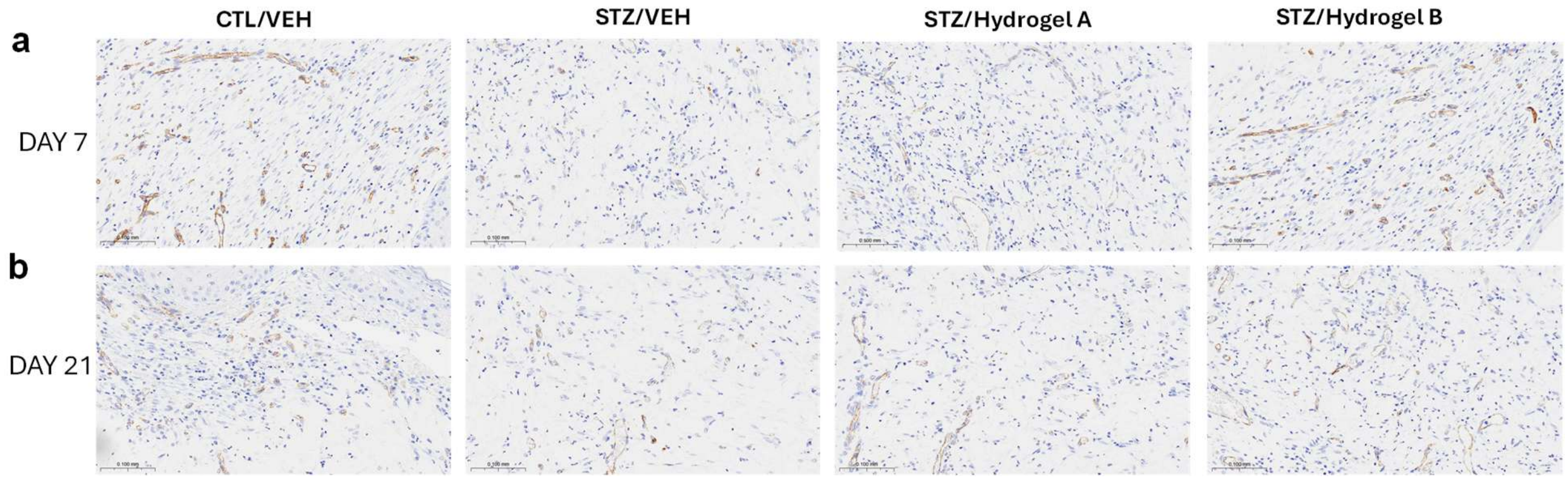

Figure S5. Representative IHC images of CD31 staining in wound tissues. Mice with cutaneous burn injuries were assigned to control (CTL) or streptozotocin-induced diabetic (STZ) groups and received topical treatment with vehicle (VEH), Hydrogel A, or Hydrogel B. Wound tissues were collected for CD31 immunohistochemical staining on day 7 (a) and day 21 (b) after burn injury.
